# Exploring the Therapeutic Mechanism of *Desmodium styracifolium* on Oxalate Crystal-Induced Kidney Injuries Using Comprehensive Approaches Based on Proteomics and Network Pharmacology

**DOI:** 10.3389/fphar.2018.00620

**Published:** 2018-06-13

**Authors:** Jiebin Hou, Wei Chen, Hongtao Lu, Hongxia Zhao, Songyan Gao, Wenrui Liu, Xin Dong, Zhiyong Guo

**Affiliations:** ^1^Department of Nephrology, Changhai Hospital, Second Military Medical University, Shanghai, China; ^2^Department of Naval Aeromedicine, Faculty of Naval Medicine, Second Military Medical University, Shanghai, China; ^3^School of Pharmacy, Second Military Medical University, Shanghai, China

**Keywords:** oxalate crystal, kidney injury, network pharmacology, proteomics, *Desmodium styracifolium*

## Abstract

**Purpose:** As a Chinese medicinal herb, *Desmodium styracifolium* (Osb.) Merr (DS) has been applied clinically to alleviate crystal-induced kidney injuries, but its effective components and their specific mechanisms still need further exploration. This research first combined the methods of network pharmacology and proteomics to explore the therapeutic protein targets of DS on oxalate crystal-induced kidney injuries to provide a reference for relevant clinical use.

**Methods:** Oxalate-induced kidney injury mouse, rat, and HK-2 cell models were established. Proteins differentially expressed between the oxalate and control groups were respectively screened using iTRAQ combined with MALDI-TOF-MS. The common differential proteins of the three models were further analyzed by molecular docking with DS compounds to acquire differential targets. The inverse docking targets of DS were predicted through the platform of PharmMapper. The protein–protein interaction (PPI) relationship between the inverse docking targets and the differential proteins was established by STRING. Potential targets were further validated by western blot based on a mouse model with DS treatment. The effects of constituent compounds, including luteolin, apigenin, and genistein, were investigated based on an oxalate-stimulated HK-2 cell model.

**Results:** Thirty-six common differentially expressed proteins were identified by proteomic analysis. According to previous research, the 3D structures of 15 major constituents of DS were acquired. Nineteen differential targets, including cathepsin D (CTSD), were found using molecular docking, and the component-differential target network was established. Inverse-docking targets including p38 MAPK and CDK-2 were found, and the network of component-reverse docking target was established. Through PPI analysis, 17 inverse-docking targets were linked to differential proteins. The combined network of component-inverse docking target-differential proteins was then constructed. The expressions of CTSD, p-p38 MAPK, and p-CDK-2 were shown to be increased in the oxalate group and decreased in kidney tissue by the DS treatment. Luteolin, apigenin, and genistein could protect oxalate-stimulated tubular cells as active components of DS.

**Conclusion:** The potential targets including the CTSD, p38 MAPK, and CDK2 of DS in oxalate-induced kidney injuries and the active components (luteolin, apigenin, and genistein) of DS were successfully identified in this study by combining proteomics analysis, network pharmacology prediction, and experimental validation.

## Introduction

The worldwide prevalence of kidney stone disease, also known as nephrolithiasis, is ~10% with a continuous increase in recent years (Scales et al., 2012; Ordon et al., 2015), endangering the public's health and patients' quality of life. Approximately 70% of crystals or stones are made of calcium oxalate (CaOx), followed by calcium phosphate, uric acid, and other salts (Liu et al., 2017; Khan et al., 2018). The damage induced by nephrolithiasis has been observed even in the earliest stage. Kidney crystals, an early form of kidney stones, have also been found to cause oxidative stress and the apoptosis of renal tubular cells (Saha et al., 2014). Therefore, the crystalline nephropathies are considered key in the prevention and treatment of obstructive nephropathy induced by kidney stones. In addition, tubular injury could also be induced by the stimulation of high oxalate in the absence of calcium (Knauf et al., 2013). Abnormal mineral metabolism, oxidative stress, inflammation, and aberrant crystallization inhibition are demonstrated to play significant roles throughout the entire process of stone formation (Taylor and Stoller, 2015). The traditional concept of kidney crystals and relative kidney injuries focuses on metabolic disturbance, which we illustrated by metabonomics approaches in our previous study (Peng et al., 2014). Recent discoveries involving the molecular mechanisms of crystal formation and crystal-induced injuries enforce a new view on this old disease and may launch novel treatment options for crystalline nephropathies. Renal tubules are the main components of the cortex and medulla junction region of kidney tissue, where the crystal mainly deposits. The present study thus aimed to characterize the molecular changes of all proteins in these predisposing locations using proteomics approaches with oxalate crystal mouse, rat, and HK-2 cell models.

Herbal remedies are increasingly being considered suitable treatments in the early-stage and long-term progression of renal stone disease. *Desmodium styracifolium* (Osb.) Merr (DS) is one of the most popular traditional Chinese herbs, and its dried aerial part has been extensively applied in the clinical therapy of renal stones. DS is confirmed to reduce CaOx deposition in the kidneys (Rodgers et al., 2014) and alleviate crystal damage through anti-inflammatory and antioxidant functions (Scales et al., 2012; Xiang et al., 2015). However, the specific molecular targets and the therapeutic mechanisms of DS are still lacking. Different constituents of DS have been extracted and analyzed in a number of experiments reported (Ma et al., 2011; Su et al., 2013). However, the active ingredient and the effective mechanism of DS in crystalline nephropathies have not been determined.

**Figure 1:**
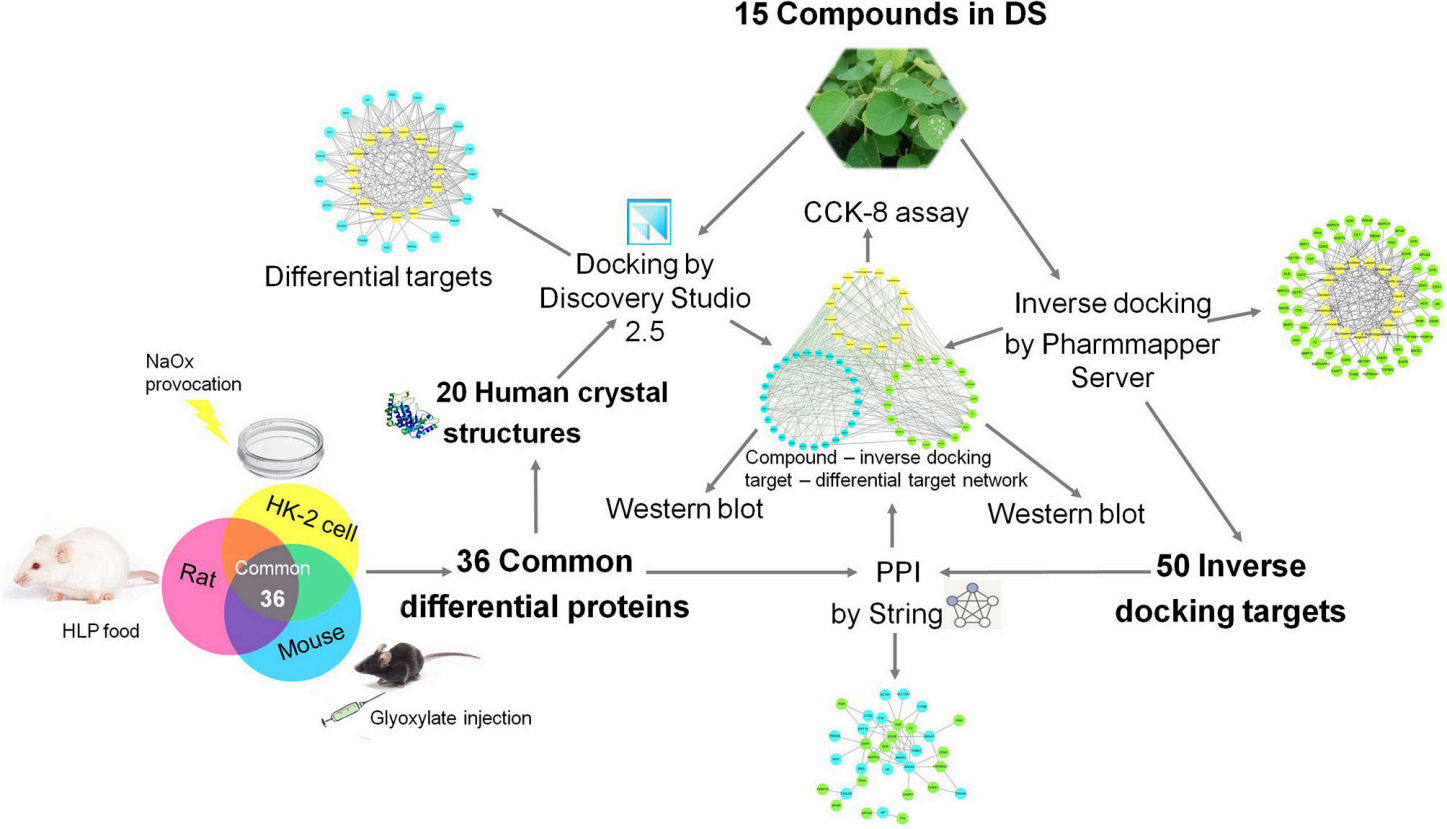
Roadmap of this study.

Due to the rapid development of bioinformatics, proteomics and network pharmacology approaches have been successfully applied in the discovery of the active components of traditional Chinese medicines and their mechanisms of action. As a popular traditional Chinese medicine, DS has an anti-nephrolithiasis effect brought about by a multi-ingredient, multi-target, multi-biological process, and multi-pathway, which coincides with the characteristics of network pharmacology (Guo et al., 2017). To uncover the specific role of DS in renal crystal injuries, we combined the proteomics approach with the network pharmacology method to explore the active components of DS and their protective molecular mechanism to clarify its medicinal value. The roadmap of this study is shown in Figure 1.

## Materials and methods

### Establishment of models

#### Cell culture

The human proximal tubular cell line, HK-2 cells were obtained from the ATCC (Manassas, USA) and maintained in DMEM-F12 supplemented with 10% FBS, 100 U/mL penicillin, and 100 mg/mL streptomycin at 37°C in 5% CO_2_. Cells were passaged upon reaching 80% confluence. Oxalate-induced kidney injury models of HK-2 cells were performed as previously described (Koul et al., 2014). Sodium oxalate (NaOx) was prepared in PBS with a pH of 7.4. The cells were randomly divided into two groups: the control group and the oxalate group with sodium oxalate at 1 mM for 12 h as the experimental conditions.

#### Experimental animals

All animal studies were performed in accordance with the National Institutes of Health (NIH) guide for the Care and Use of Laboratory Animals. The experimental procedures were approved by the Ethical Committee for the Experimental Use of Animals at Second Military Medical University (Shanghai, China).

Sixteen wild-type male C57BL/6 mice (7–8 weeks old) were purchased from Shanghai SLAC Animal Co., Ltd. (Shanghai, China). All mice had free access to drinking water and regular chow every day and were kept under a controlled 12 h light/dark cycle at 20–25°C with the relative humidity at 55–65%. After conditioned housing for 1 week, 16 mice were equally divided into the control group and the oxalate model group, with eight mice each. The oxalate group was administered glyoxylate (100 mg/kg/day) by daily intra-abdominal injections for 7 consecutive days, and the control group was intra-abdominally injected daily with normal-volume saline (20 mL/kg/day) per day. On day seven after the administration of glyoxalate, kidney samples were collected, and the cortex and medulla junction tissue were dissected to be further analyzed.

Sixteen male Sprague-Dawley rats (120–130 g) at the age of 5 weeks were purchased from Shanghai SLAC Laboratory Animal Co., Ltd (Shanghai, China). Three rats per cage were maintained under standard laboratory conditions (a temperature of 20–25°C, relative humidity of 55–65%, and 12 h/12 h light/dark cycle) with aseptic food and tap water *ad libitum*. After 1 week of habituation, the rats were randomized according to their weight into the control and oxalate model groups, with eight rats in each group. A model of oxalate kidney stones was established by the administration of food, which was supplemented with 5 g/kg dose of hydroxyproline per day; the control group received the same volume of 0.5% CMC-Na. No animals died or showed severe clinical symptoms before reaching the 28 days of treatment. At the end of 28 days, the rats were sacrificed and both kidneys of the rats were excised. After removing the capsule and pelvis, kidney samples were collected, and the cortex and medulla junction tissues were dissected for further analysis.

#### Protein extraction

For protein extraction, cells and the cortex and medulla junction tissue from rats or mice were respectively homogenized and lysed in lysis buffer (KeyGEN BioTECH, China). Then, the homogenate was centrifuged at 13,000 r/min for 10 min at 4°C, and the supernatant, which contained proteins, was recovered. The protein concentrations in each sample were measured using a BCA kit (Thermo Fisher Scientific) according to the manufacturer's protocol.

### Proteomics analysis

#### iTRAQ-labeling

After the concentration determination, equal aliquots from protein samples in the same group were mixed together. A 120 μL portion of the pooled protein sample from each group was transferred into one tube and reduced with TCEP, alkylated with MMTS, and digested with trypsin at room temperature according to the iTRAQ (isobaric tags for relative and absolute quantitation) reagent reference guide (AB Framingham, MA, Sciex, USA). The obtained tryptic peptides from the control and oxalate groups were respectively labeled with iTRAQ reagents with different tags for 2 h at room temperature and then terminated by adding H_2_O to 30% of the total reaction volume to inactivate the iTRAQ regents.

#### Mass spectrometry analysis

The total proteins of mice and HK-2 cells were analyzed based on Matrix-assisted laser desorption/ionization-time of flight-mass spectrometry (MALDI-TOF-MS). The dried pooled peptides were then reconstituted in A phase (20 mM ammonium formate solution, pH 10) for high-pH reverse-phase liquid chromatography (RPLC) separation, using a Shimadzu LC−20AB HPLC system with an Ultremex SCX column (250 × 4.6 mm), with an 0.8 mL/min flow rate. Fractions were collected and combined into new fractions according to parameters. MALDI-TOF-MS was used for protein identification. The combined fractions were mixed with MALDI matrix (2 mg/mL-cyano-4-hydroxycinnamic acid in CH3OH: isopropanol: CH3CN:H2O:acetic acid (12:33.3:52:36:0.7, v/v/v/v) containing 10 mM ammonium phosphate). The matrix was delivered with a PHD200 infusion pump (Harvard Apparatus) at 0.8 L/min. Fractions were deposited at 30-s intervals on a stainless steel MALDI target plate (1536 spots/plate; Applied Biosystems). The MS was operated in positive ion mode across the mass range of 800–1250 m/z using a 5800 TOF/TOF spectrometer (Applied Biosystems/MSD Sciex). The scan of MS/MS was completed in a data-dependent manner, and the most abundant 15 peaks were selected in per spot with a minimum signal-to-noise ratio of 40. The atmosphere was used as collision gas with collision energy of 1 kV. The proteins of rats were analyzed using LC-MS/MS analysis, and the detailed method is described in the Supplementary Material. The proteomics data have been deposited to the ProteomeXchange Consortium via the PRIDE partner repository with identifier PXD009496.

#### Data processing for proteomics

All MS/MS data were acquired with a data converter of AB sciexms (version 1.3). The acquired peak-lists of all MS/MS spectra were searched with Mascot (Matrix Science, London, UK; version 2.5.1) against the corresponding mouse, rat or human database downloaded from Uniprot Knowledgebase (http://www.uniprot.org/). For data integration and validation, the search results were input into Scaffold Q+ software (version Scaffold_4.6.1, Proteome Software Inc., Portland, OR). The peptide and protein false-discovery rate (FDR) were both set to ≤1%, and protein identifications were accepted if they contained at least two identified peptides and at >99% probability. For quantitation analysis, Scaffold Q^+^ was used to quantify the isobaric tag peptide and protein identifications. Mann-Whitney *U*-tests for each group were calculated using SPSS 20.0, and *p* < 0.05 was considered statistically significant. In this experiment, proteins with a concentration ratio (oxalate/control) > 2.0 and < 0.5 were considered the significantly differential expression proteins.

### Pharmacology analysis

#### Constituent compounds of DS

Information on the main compounds in DS was acquired according to previous research (Zhou et al., 2012; Su et al., 2013). The most popular constituent compounds of DS were selected according the detailed references listed in the Supplementary Materials. The acquired 15 compounds (i.e., 2′-hydroxygenistein, apigenin, aromadendrin, chrysoeriol, formononetin, genistein, homoferreirin, isoschaftoside, isovitexin, luteolin, schaftoside, vanillic acid, vicenin-2, vicenin-3, and β-sitosterol) were further searched in Pubchem (https://pubchem.ncbi.nlm.nih.gov) to get the 3D molecular structure of each compound saved in “SDF” file format.

#### Molecular docking of differential proteins

The differential proteins acquired from proteomic analysis were considered potential proteins related to crystal-induced kidney injuries. The human structures of these differential proteins were collected from the protein data bank (PDB) (http://www.rcsb.org) as potential targets for docking. The whole work of docking was conducted using the commercial software Discovery Studio 2.5 (http://www.accelrys.com). First, the X-ray crystal structures of protein targets were preprocessed. Hydrogen was added to the model, and its orientation was optimized using the CHARMm force field energy minimization while all non-hydrogen atoms were not allowed to move. Each protein was defined as a receptor, and the proteins' active sites were found from the receptor cavities using the Discovery Studio tool. Then, the docking protocol was performed to show the interaction of components in DS with the differential proteins using LibDock. As Libdock can provide 10–100 predicted dockscores from different docking poses for each compound in a binding pocket of a protein, we only considered the best dockscore. The cutoff value of the dockscore for differential targets was set as 80 in this protocol.

#### Prediction and screening of inverse-docking targets

Given that compounds in DS may act on these differential proteins in an indirect way, the acquired 15 component structures were imported into PharmMapper Server (http://lilab.ecust.edu.cn/pharmmapper/, updated in Jan 1st, 2016), which is an updated integrated pharmacophore matching platform for the identification of potential targets with inverse-docking approaches (Wang et al., 2017). The species of the acquired targets was limited to “Homo sapiens.” We selected the top 10 inverse-docking targets for subsequent study. Because of the nonstandard naming, we obtained their official symbols using UniProtKB, which is the central hub for the collection of functional information on proteins with accurate, consistent, and rich annotation.

#### Protein–protein interaction (PPI)

To explore the indirect effect of DS on the differential proteins, the PPI between potential inverse-docking targets from PharmMapper and the differential proteins were analyzed by String (http://string-db.org/, ver. 10.5), which is a database of known and forecasted PPIs, with the species limited to “Homo sapiens” and a confidence score >0.4. Each node represents proteins produced by a single protein-coding gene locus.

#### Network

To facilitate the scientific interpretation of complex relationships between active components of DS and nephrolithiasis-related protein targets, the network was generated by Cytoscape (http://www.cytoscape.org/), which is an open-source software project for integrating biomolecular interaction networks with high-throughput expression data into a unified conceptual framework.

### DS treatment and protein targets

#### Preparation of DS samples

DS was purchased from Tongrentang Co., Ltd (Shanghai, China). The dried aerial part of the DS plant was pulverized to powder by a disintegrator. The powder (250 g) was extracted with 1 L of 75% ethanol for 2 h by an ultrasonic method at room temperature. The extraction solution was filtrated followed by concentration using a rotary evaporator at 55°C to dryness. The yield of the ethanol extraction was 12.7% (w/w). The extract powder was dissolved in saline solution to obtain a concentration of 50 mg/ml for the following experiments.

#### DS treatment of animals

Twenty-four wild-type male C57BL/6 mice (7–8 weeks old) were purchased from the Cavens (Changzhou, China). After conditional housing for 1 week, these mice were randomly divided into four experimental groups including a control group, an oxalate group, a DS low-dose group, and a DS high-dose group with six mice in each group. To establish the crystal renal injury model, the mice were intraperitoneally injected with glyoxylate at a dosage of 100 mg/kg once daily for 7 days, except for the control group, which was injected with saline. After each glyoxylate injection, the mice in the two DS groups were respectively given the DS extract at a low dose of 10 mL/kg (equivalent to 500 mg/kg body weight of the extract dried powders) and a high dose of 20 ml/kg by gastric perfusion. Mice from the control and oxalate groups were i.g. administered saline.

On day 7 after the administration of glyoxalate, blood was collected from the orbital sinus, and the serum sample was separated by centrifugation at 4,000 rpm at 4°C for 5 min. The supernatant was aliquoted into sterile tubes and frozen at −80°C. After urine and blood collection, bilateral kidney tissue samples from 24 mice were harvested. Sections of the right kidneys were fixed in neutral buffered formalin (10%) for further analysis. The left kidneys were dissected and frozen at −80°C for future use.

#### Histological and biochemical analysis

The fixed kidney samples were further embedded in paraffin and sectioned at a thickness of 3 μm. After staining with the Von Kossa method according to the instructions of the commercial kit (Shunbai, Shanghai, China), images of randomly selected areas from each section were collected by a light microscope. Twenty views were gathered from each group for semi-quantitative examination by Image J software (https://imagej.nih.gov/ij/). Some remaining sections were colored with the hematoxylin-eosin for evaluation under an optical microscope.

The levels of calcium (C004-1) and 8-hydroxydeoxyguanosine (8-OHdG) (H165) in kidney tissue and the urinary ratio of calcium to creatinine (Ca/Cr) (C011-1) were respectively measured using a corresponding commercial kit (Jiancheng, Nanjing, China). The concentration of the serum excretion of kidney injury molecule-1 (KIM-1) was tested using an ELISA kit (USCN, Wuhan, China; SEA785Mu).

### Active compounds and cell culture

#### Cell culture and treatment

HK-2 cells were maintained in DMEM-F12 supplemented with 10% FBS, 100 U/mL penicillin, and 100 mg /mL streptomycin at 37°C in 5% CO_2_. As previously described, oxalate-induced kidney injury models of HK-2 cells were established with NaOx at 1 mM for 12 h as the experimental conditions. Luteolin, apigenin, and genistein were obtained from MCE (Shanghai, China) and were resuspended in DMSO to be used at indicated concentrations. HK-2 cells in treated groups were co-incubated for 12 h with various concentrations of luteolin, apigenin, or genistein with NaOx.

#### CCK-8 assay

Cell proliferation was monitored using a Cell Counting Kit-8 (CCK-8) (Beyotime Shanghai, China). In brief, HK-2 cells were suspended in medium with or without NaOx stimulation and compound treatment and then plated in 96-well plates at a 2000 cells/well concentration. Cell proliferation was measured after adding CCK-8 reagent 1 h at a 450 nm absorbance.

### Quantitative real-time PCR analysis

Total RNA was acquired from HK-2 cells using TRIzol reagent (Invitrogen, USA). The absorbances at 260 and 280 nm were measured to confirm the quantity and purity of total RNA. cDNA was synthesized from total RNA using reverse transcriptase M-MLV (RNase H-) (Takara, Japan). SYBR Green Master Mix (YESEN, China) was used to measure the relative expression levels of mRNA. All the primers were designed and synthesized by Sangon, Shanghai, China, and the sequences of primers are listed in Supplementary Table S3. The relative expression levels of targeting genes were normalized to β-actin gene and calculated using the 2^ΔΔ*CT*^ method.

### Western blot

Acquired cells from three repeated experiments and corticomedullary tissues from 24 mice were homogenized in lysis buffer (KeyGEN, Nanjing, China) containing protease inhibitor and phosphatase inhibitor. The lysates were centrifuged at 12,000 rpm for 5 min at 4°C and the supernatant was collected. The protein concentration of mixed lysates was determined using a BCA protein assay kit (Thermo Fisher Scientific). Equal amounts of total protein were subjected to SDS-PAGE gel for separation and transferred onto a nitrocellulose membrane (GE Healthcare Life Sciences). After blocking, the membrane was incubated with rabbit polyclonal anti-CTSD (1:1,000, Abways), anti-phospho-CDK2 (Thr160) (1:1,000, Abways), and anti-phospho-MAPK 14(Thr180/Tyr182) (1:1,000, Abways) antibodies at 4°C overnight. After washing with TBST, the membrane was incubated with a fluorescence-conjugated secondary anti-rabbit antibody (1:10,000, Licor) for 60 min at room temperature. The signals were visualized using the Odyssey Infrared Imaging System (Licor, NE, USA) and quantitatively analyzed by normalizing them to β-actin using the Image J software.

### Statistical analysis

Data are expressed as mean ± SEM and were plotted in histograms with GraphPad Prism 5.0. Statistical analysis was performed using a one-way analysis of variance (ANOVA) or the Kruskal-Wallis H test as appropriate to assess differences among groups. Bonferroni's test or the Mann-Whitney *U*-test was further employed for post-hoc comparisons. The significance level was set at *p* < 0.05.

## Results

### Proteomics study

Supplementary Figure S1 showed that 114 proteins of HK-2 cells, 197 proteins of mice, and 201 proteins of rats were statistically differentially expressed between the oxalate model group and the control group. In the present study, the differentially expressed proteins that are included in all models (HK-2 cell, mouse, and rat models) with the same change trends were selected as common differential proteins for further analysis. Thirty-six common differential proteins were finally acquired and are listed in Table 1, among which 20 proteins were up-regulated (>2.0-fold) and 16 were down-regulated (<0.5-fold).

**Table 1:** Common differential proteins in the oxalate group compared to the control group.

**No**.	**Protein name**	**Gene**	**Rat**		**Mouse**		**HK-2**	
			FC^*^	***P*-Val**	**FC**	***P*-Val**	**FC**	***P*-Val**
1	14-3-3 protein eta	Ywhah	4.57	0.000	2.03	0.013	3.01	0.000
2	A-kinase anchor protein 12	Akap12	6.79	0.000	4.45	0.000	2.79	0.000
3	Alpha-actinin-1	Actn1	5.55	0.000	2.38	0.000	4.28	0.000
4	Annexin A1	Anxa1	3.66	0.000	2.25	0.000	2.98	0.000
5	Annexin A2	Anxa2	2.78	0.000	2.38	0.000	2.44	0.000
6	Annexin A3	Anxa3	2.58	0.003	4.79	0.027	3.84	0.000
7	Annexin A5	Anxa5	3.50	0.000	2.38	0.001	3.02	0.000
8	Cathepsin D	Ctsd	7.87	0.000	3.91	0.039	6.77	0.000
9	Collagen alpha-1(XII) chain	Col12a1	2.56	0.000	4.97	0.000	2.34	0.001
10	C-type mannose receptor 2	Mrc2	3.53	0.001	2.58	0.009	2.46	0.000
11	Cytoglobin	Cygb	2.96	0.000	2.27	0.023	2.33	0.007
12	Cytoskeleton-associated protein 4	Ckap4	2.17	0.007	2.17	0.000	2.44	0.000
13	Extended synaptotagmin-1	Esyt1	2.36	0.000	2.54	0.000	2.12	0.008
14	Fibronectin	Fn1	3.77	0.000	2.63	0.000	3.02	0.000
15	Haptoglobin	Hp	9.82	0.000	10.96	0.000	8.03	0.000
16	Keratin, type I cytoskeletal 19	Krt19	2.40	0.028	3.94	0.000	3.55	0.000
17	Major vault protein	Mvp	2.81	0.000	2.49	0.000	2.11	0.022
18	Thrombospondin-1	Thbs1	8.79	0.000	3.80	0.000	6.19	0.000
19	Transcription intermediary factor 1-beta	Trim28	4.13	0.000	2.51	0.037	2.79	0.000
20	Transgelin-2	Tagln2	2.11	0.000	2.58	0.002	2.33	0.000
21	Basigin	Bsg	0.17	0.000	0.46	0.038	0.38	0.000
22	Cystathionine gamma-lyase	Cth	0.24	0.000	0.37	0.001	0.43	0.000
23	Cysteine sulfinic acid decarboxylase	Csad	0.37	0.002	0.37	0.000	0.31	0.000
24	Glycerol kinase	Gk	0.42	0.001	0.40	0.000	0.47	0.011
25	Homogentisate 1,2-dioxygenase	Hgd	0.36	0.000	0.45	0.000	0.38	0.000
26	Inositol oxygenase	Miox	0.45	0.001	0.45	0.000	0.04	0.000
27	LETM1 and EF-hand domain-containing protein 1, mitochondrial	Letm1	0.13	0.000	0.44	0.000	0.27	0.000
28	Meprin A subunit alpha	Mep1a	0.08	0.000	0.40	0.011	0.38	0.007
29	Methylcrotonoyl-CoA carboxylase beta chain, mitochondrial	Mccc2	0.50	0.000	0.42	0.000	0.44	0.013
30	MICOS complex subunit Mic60	Immt	0.28	0.000	0.49	0.000	0.35	0.000
31	Short-chain specific acyl-CoA dehydrogenase, mitochondrial	Acads	0.46	0.033	0.37	0.000	0.42	0.001
32	Sodium/glucose cotransporter 2	Slc5a2	0.14	0.000	0.39	0.000	0.19	0.000
33	Sodium/potassium-transporting ATPase subunit beta-1	Atp1b1	0.45	0.001	0.35	0.000	0.39	0.000
34	Sodium-dependent neutral amino acid transporter B(0)AT1	Slc6a19	0.14	0.001	0.47	0.013	0.31	0.008
35	Solute carrier family 12 member 1	Slc12a1	0.30	0.000	0.35	0.000	0.27	0.000
36	Solute carrier family 22 member 12	Slc22a12	0.10	0.000	0.49	0.004	0.27	0.000

* *Fold change. The ratio of relative amounts of oxalate group to control group*.

### Direct relationship between DS and differential proteins

**Figure 2:**
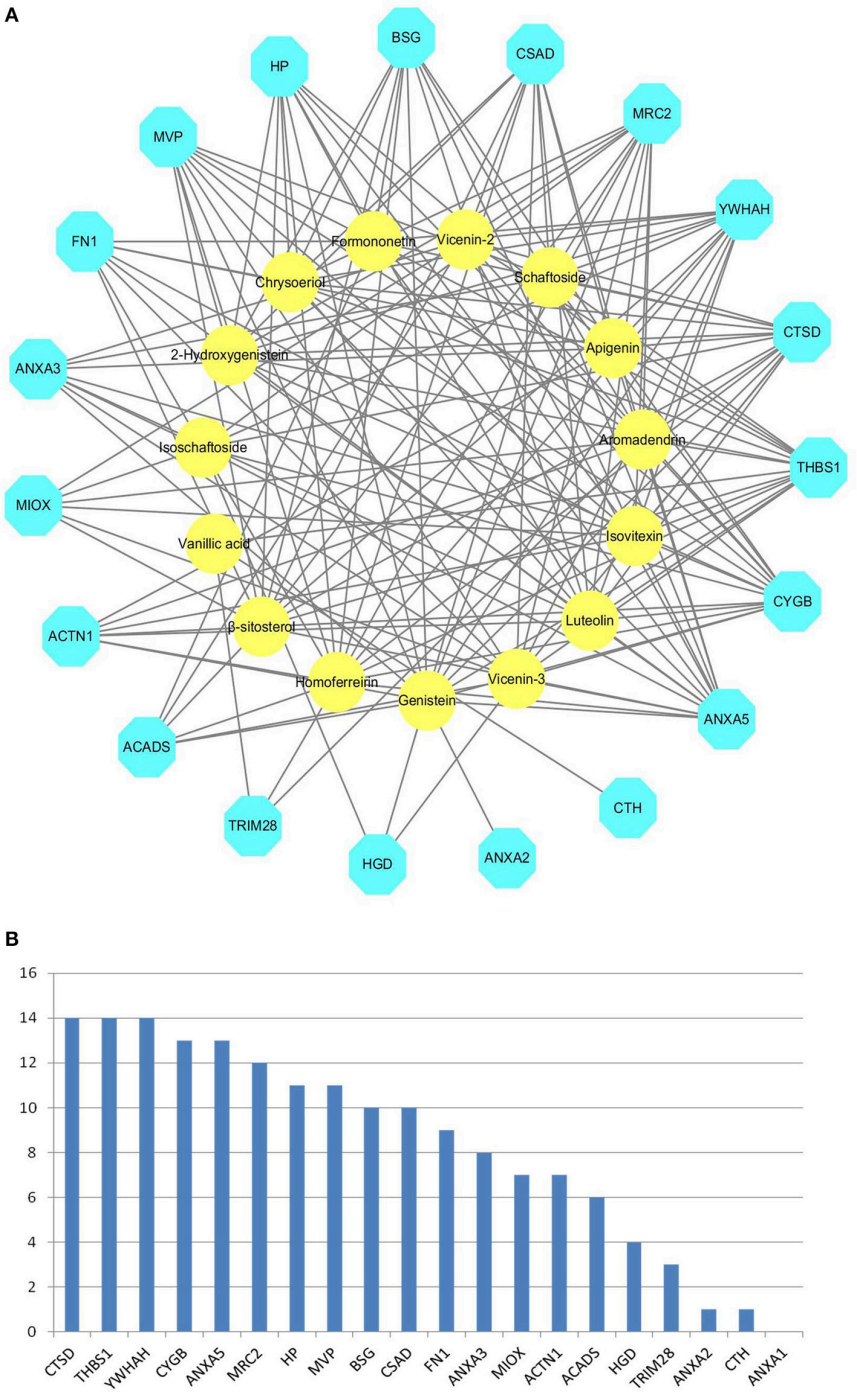
Compound-differential target network. **(A)** The network of 15 compounds predicted to have 19 potential differential protein targets. The yellow circles represent the compounds, while the blue octagons delineate the proteins. **(B)** The numbers of compounds docked with differential targets.

We obtained 20 X-ray crystal structures of differential proteins from PDB as potential targets for the docking analysis of the 15 main components in DS. The detailed docking results are listed in Supplementary Table S1. A network analysis was performed by connecting compounds and their docked differential targets with docking scores higher than the 80 cutoff value. Thus, we can roughly observe the relationships between active compounds and targets from the compound-differential target network (Figure 2A). This network contains 34 nodes (19 differential target nodes and 15 compound nodes) and 165 edges. The numbers of compounds docked with differential targets are listed in Figure 2B. CTSD, THBS1, and YWHAH can be controlled by as many as 14 compounds. Genistein can regulate the most targets. This suggests that compounds of DS may directly act on these differential proteins synergistically, showing the herbal drug's multi-compound to multi-target feature.

### Indirect relationship between DS and differential proteins

#### Network of the compound-inverse docking target

**Figure 3:**
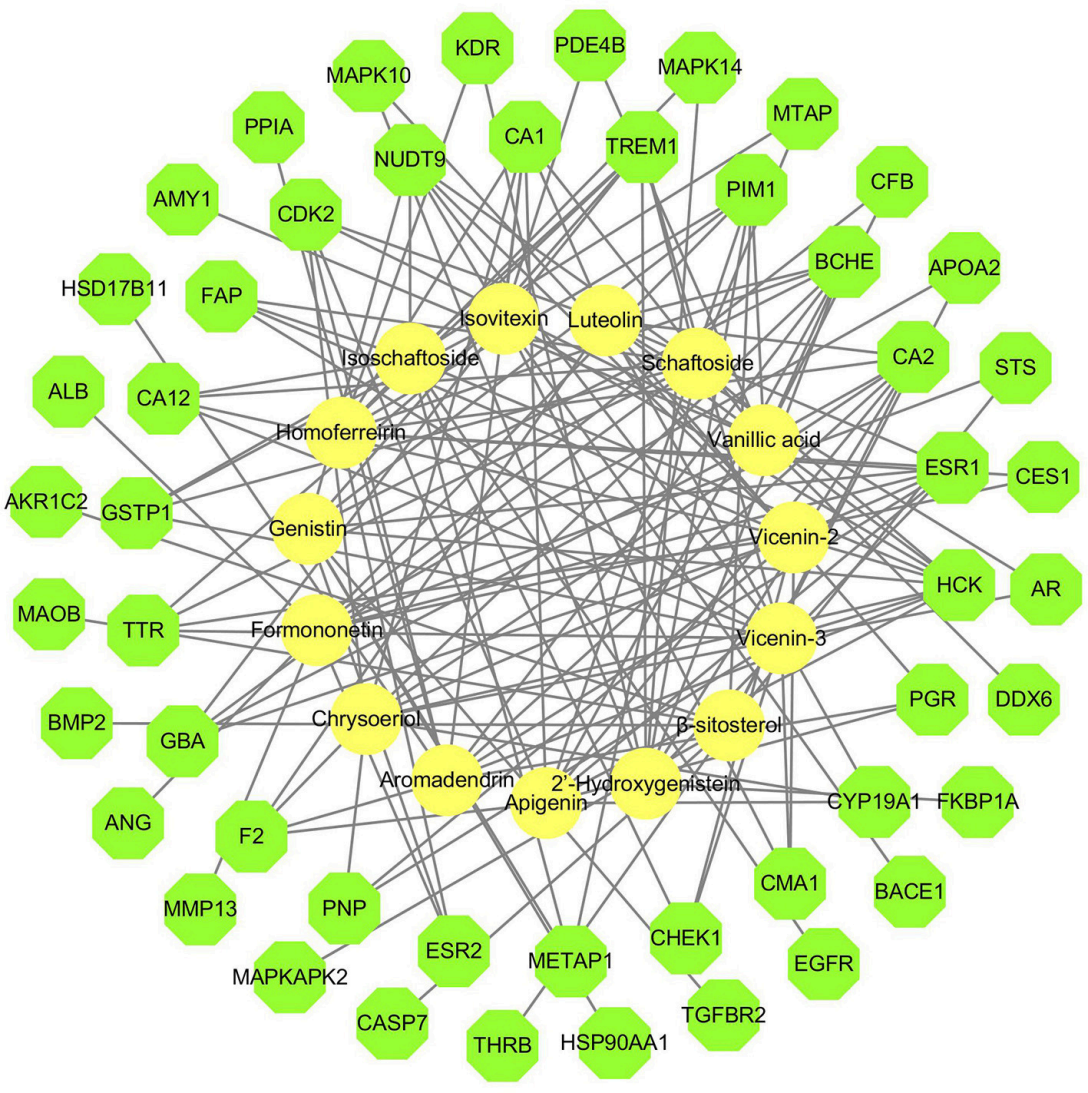
Compound-inverse docking target network. The network of 15 compounds predicted to have 50 inverse-docking targets. The yellow circles represent the compounds, while the green octagons delineate the proteins.

DS may also indirectly affect these nephrolithiasis-related differential proteins through other protein targets. Information on more potential targets was obtained by performing inverse docking by PharmMapper. The details of the top 10 targets are described in Supplementary Table S2, and 50 inverse-docking targets were acquired in total. The compound-inverse docking target network was constructed in Figure 3; nodes close to the center show more interactions with compounds than peripheral nodes. This indicates that some central targets including CDK2 were hit by numerous compounds. However, peripheral nodes including mitogen-activated protein kinase-14 (MAPK14), usually known as p38 MAPK, could only be modulated by two compounds.

#### PPI network

**Figure 4:**
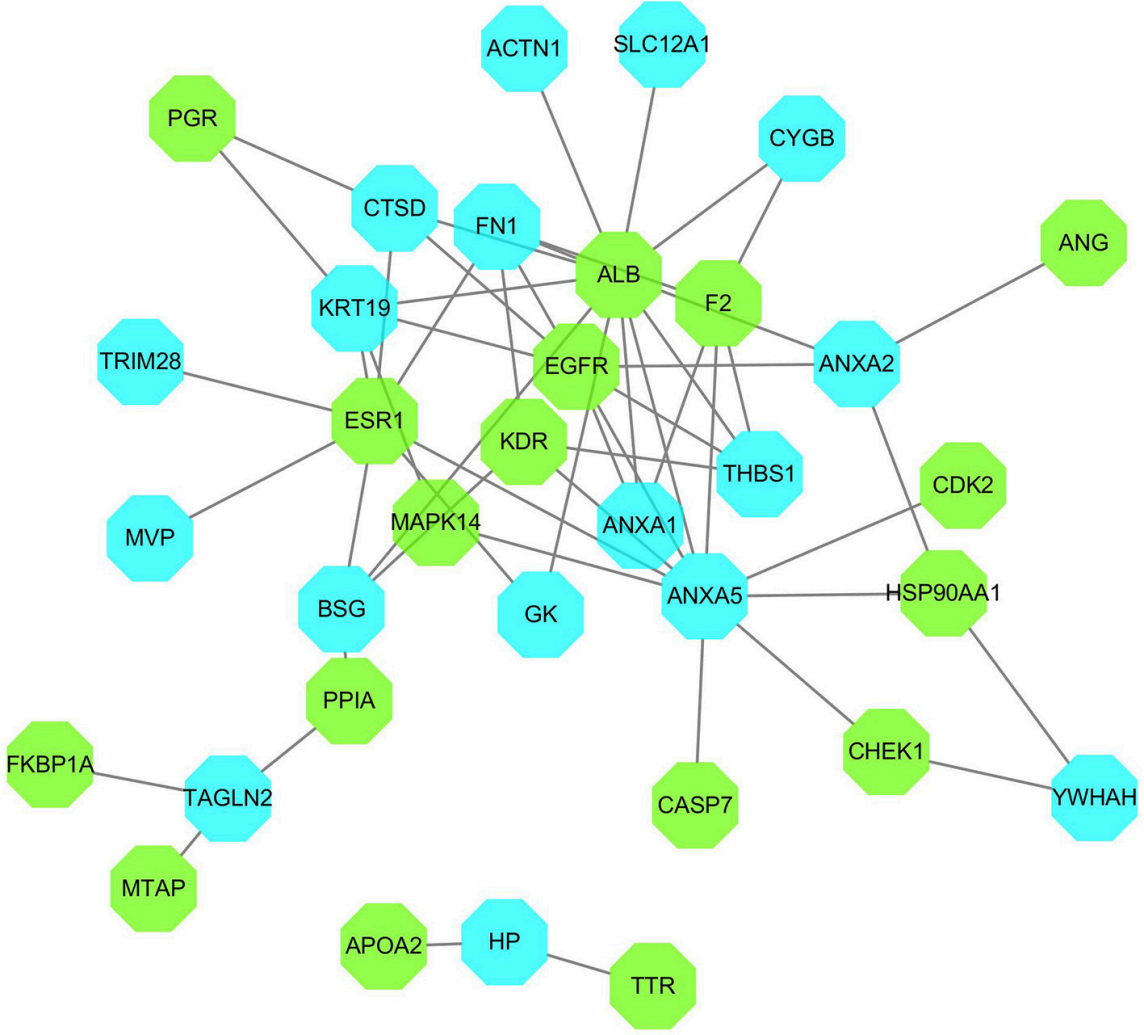
PPI network of inverse-docking targets and differential proteins. The blue octagons represent the differential proteins, and the green octagons represent the inverse-docking targets.

The differential proteins and the inverse-docking targets were imported into String together to be analyzed based on the PPI (Supplementary Figure S2). The internal relationships within inverse-docking targets were not considered, and the nodes of inverse-docking targets, which have no interaction with the differential proteins, were removed in the constructed PPI network (Figure 4), where 17 inverse-docking targets were found to interact with 17 differential proteins.

### Integrated network of direct and indirect relationships

**Figure 5:**
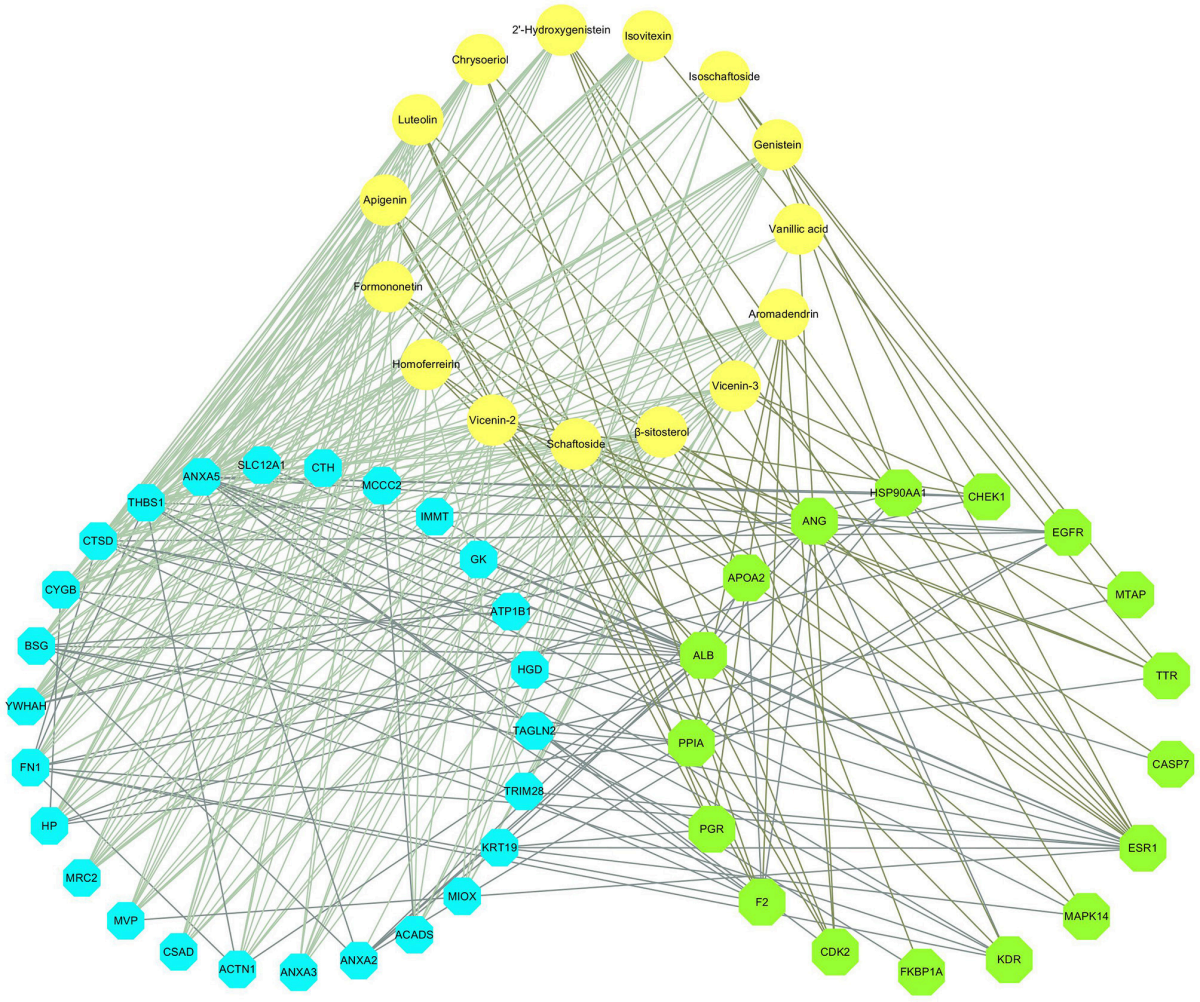
Compound-inverse docking target-differential protein network. The yellow circles represent the compounds. The green octagons delineate the inverse-docking targets, and the blue octagons delineate the differential proteins.

The compound-differential target network was combined with the compound- inverse docking target network and PPI network to establish an integrated compound-inverse docking target-differential protein network (Figure 5). This integrated network is composed of 59 nodes (15 compound nodes, 17 inverse docking target nodes, and 26 differential protein nodes) and 281 edges. The direct and indirect relationships between DS and crystalline nephropathy related differential proteins are both showed in this network, indicating multiple potential therapeutic effects of DS on nephrolithiasis.

### Calcium deposition and renal injuries

**Figure 6:**
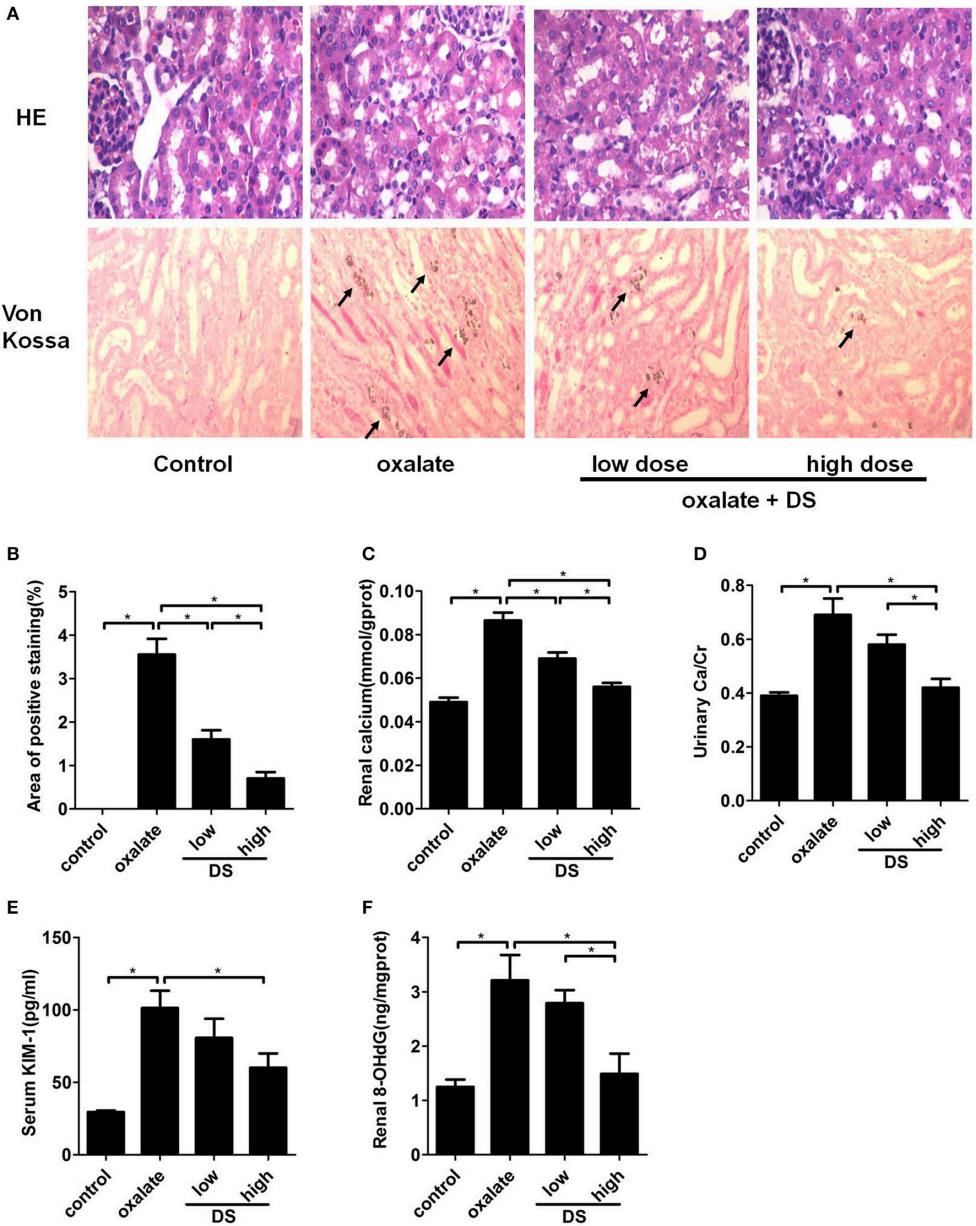
Histological and biochemical analysis of the mouse model induced by oxalate. **(A)** Representative images for the HE staining and Von Kossa staining of calcium deposition in the cortex and medulla junction of the kidneys. **(B)** Semi-quantitative analysis of calcium deposition in the area of positive staining from every 20 random views. The levels of renal calcium **(C)**, urinary Ca/Cr **(D)**, serum KIM-1 **(E)**, and renal 8-OHdG **(F)**. ^*^P < 0.05.

The representative images of Von Kossa calcium staining for each group are illustrated in Figure 6A, showing that CaOx deposits were clearly present on the renal corticomedullary junction in the mice in the oxalate group. In addition, the level of positive staining for CaOx deposits in the renal sections from the DS-treated group at both doses was much less than that in the oxalate group. Semi-quantitative analysis confirmed a significant difference in the positive staining areas of calcium deposition among different groups (Figure 6B).

The concentration of calcium in renal tissue and the urinary Ca/Cr increased significantly after the injection of glyoxylate, and this increased trend was significantly reversed by the administration of DS, especially at a high dose (Figures 6C,D). For the renal injury evaluation, the serum KIM-1, and renal 8-OHdG levels were significantly lower in the DS high dose-treated groups than those of the oxalate group (Figures 6E,F), supporting the protective effect of DS on oxalate-induced renal injuries. The treatment of DS at a high dose exerts a better therapeutic effect than at a low dose.

### Validation of potential targets

**Figure 7:**
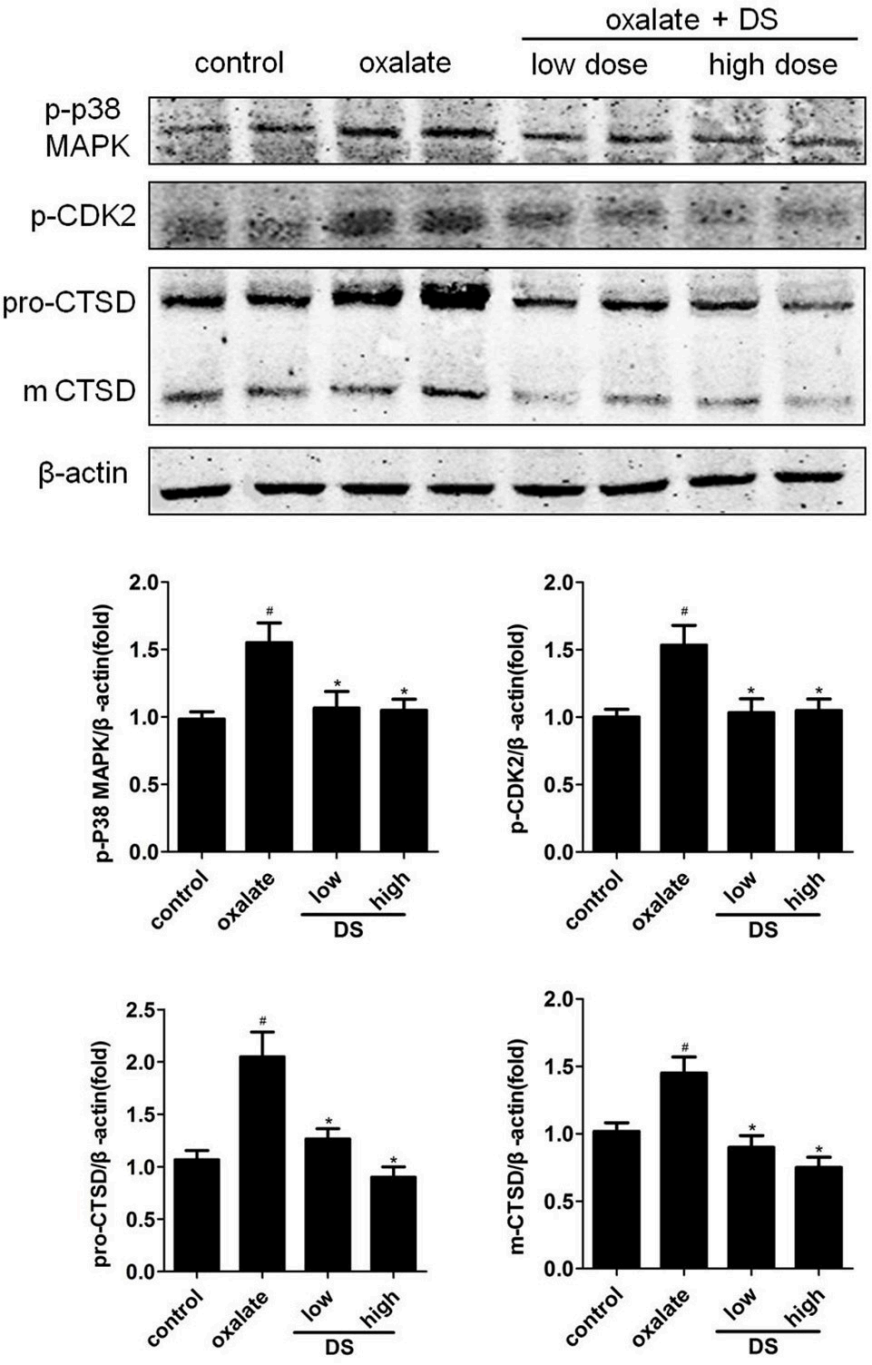
Effects of DS on the level of CTSD, p-CDK2, and p-p38 in mice. The western blotting results of CTSD, p-CDK2, and p-p38 in the corticomedullary region of the kidneys. Data were expressed as mean ± SEM (*n* = 6). ^#^*P* < 0.05 vs. the control group, ^*^P < 0.05 vs. the oxalate group.

The effects of DS on both potential differential targets and inverse-docking targets were further investigated based on a mice model. As illustrated in Figure 7, the level of CTSD was significantly increased in the oxalate group and significantly reduced by DS treatment at both doses in the corticomedullary region of kidney tissue. For potential inverse-docking targets, significantly increased level of p-p38 MAPK and p-CDK2 was investigated in the oxalate group, and these induced phosphorylation levels of p-p38 MAPK and p-CDK2 were significantly decreased under the treatment of DS. However, the differential expression of p-p38 MAPK and p-CDK2 is not obvious between DS groups with different doses.

### Active components of DS

**Figure 8:**
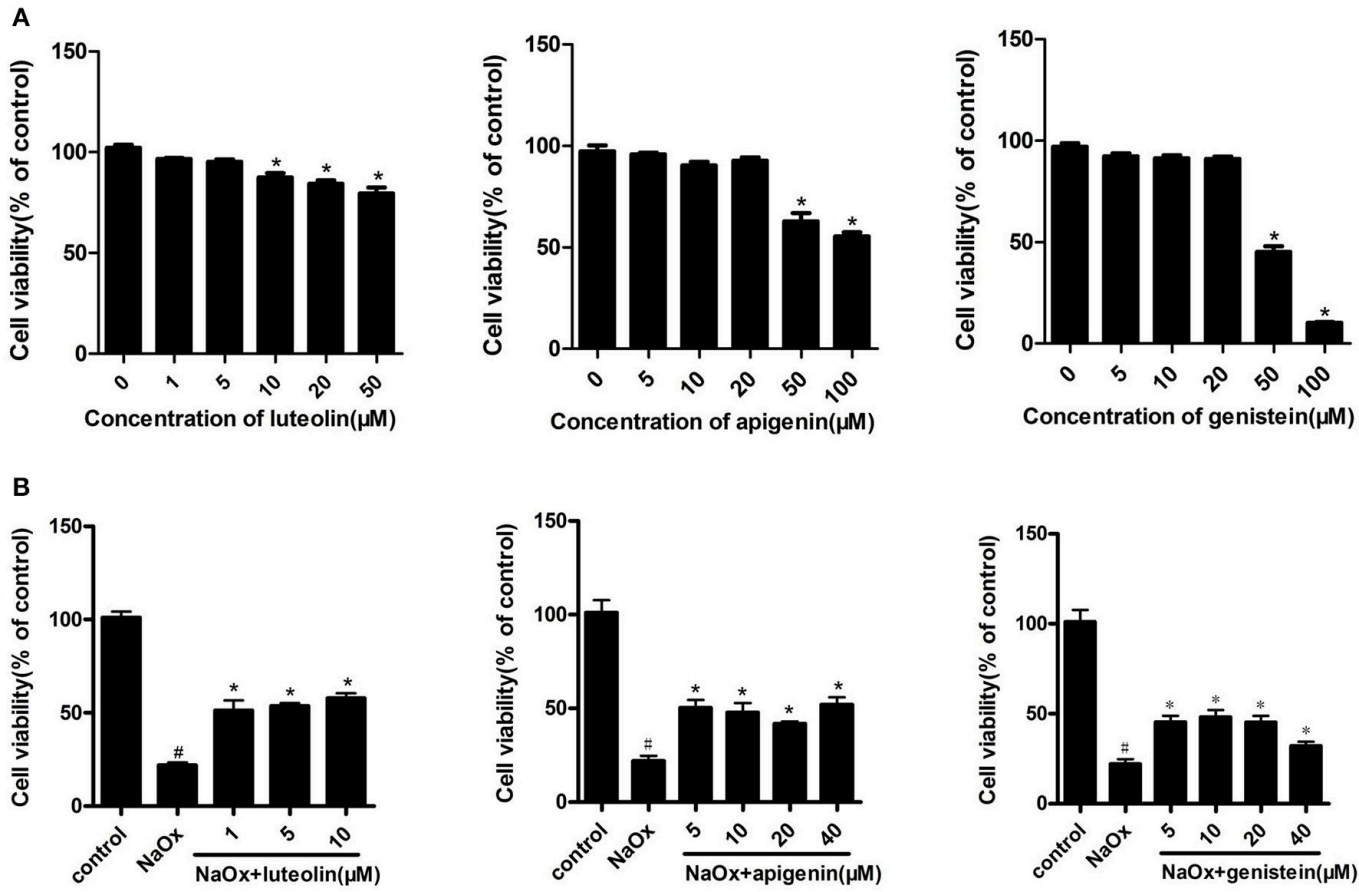
The effects of luteolin, apigenin, and genistein on cell viability in oxalate-stimulated HK-2 cells. **(A)** Effects of luteolin, apigenin, and genistein on cell viability in HK-2 cells. Cells were incubated for 12 h with different concentrations of luteolin, apigenin, and genistein. ^*^P < 0.05 vs. untreated cells. **(B)** The effects of luteolin, apigenin, and genistein on cell viability in oxalate-stimulated HK-2 cells. Oxalate-stimulated cells were incubated for 12 h with NaOx (1 mM) and different concentrations of luteolin, apigenin, and genistein. ^#^*P* < 0.05 vs. untreated cells; ^*^P < 0.05 vs. cells treated with NaOx alone.

HK-2 cells were incubated with different concentrations of luteolin, apigenin, and genistein for indicated times, and their viability was determined using an CCK-8 assay. After incubation with 10 μM of luteolin, 50 μM of apigenin, or 50 μM of genistein for 12 h, their cell viability was significantly reduced. The effects of luteolin, apigenin, and genistein on the cell viability of oxalate-induced kidney injury models of HK-2 cells were further evaluated. The results showed that luteolin, apigenin, and genistein had a clear protective effect on the cell viability of NaOx-treated cells at the indicated concentrations (Figure 8).

The molecular mechanisms of the protective effects of luteolin, apigenin, and genistein on NaOx-treated HK-2 cells were studied. As shown in Supplementary Figure S3, the significantly increased mRNA levels of CTSD, THBS1, and ANXA5 in HK-2 cells induced by NaOx decreased significantly with the co-incubation of luteolin, apigenin, and genistein.

**Figure 9:**
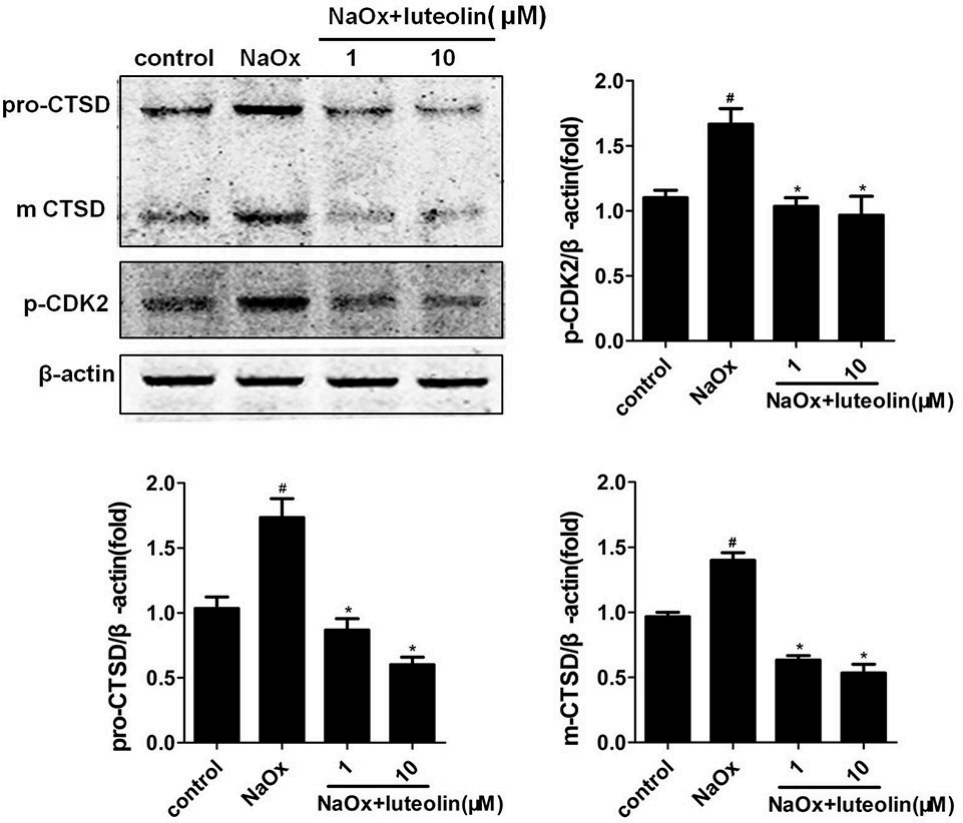
Effects of luteolin on the level of CTSD and p-CDK2 in HK-2 cells. The western blotting results of CTSD and p-CDK2 in the NaOx-stimulated HK-2 cells treated by luteolin. Data were expressed as mean ± SEM (*n* = 3). ^#^*P* < 0.05 vs. untreated cells; ^*^P < 0.05 vs. cells treated with NaOx alone.

Then, the effects of luteolin on the expression of the potential protein targets were further investigated. As depicted in Figure 9, the co-incubation with luteolin led to a significant inhibition of the NaOx-induced production of CTSD in HK-2 cells. Luteolin also significantly reduced the induced phosphorylation of NaOx-stimulated HK-2 cells.

## Discussion

**Figure 10:**
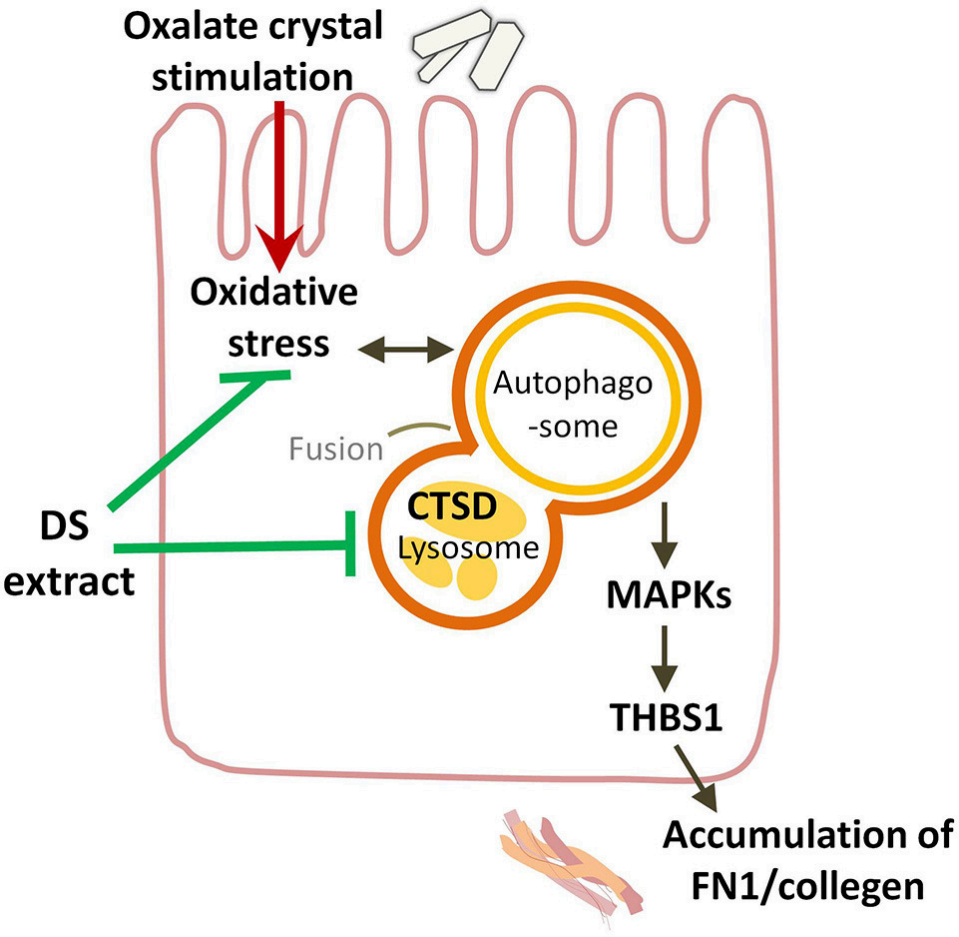
Overview of potential mechanisms underlying the protective effects of DS on oxalate-induced renal injuries. DS could attenuate the renal injury induced by oxalate crystals through suppressing the autophagic activity and the renal fibrogenesis.

As shown in recent studies, the excessive activation of autophagy plays an important role in crystalline nephropathy (Duan et al., 2018; Liu et al., 2018). The exposure of HK-2 cells to CaOx crystals increased the autophagic activity, and the inhibition of autophagy attenuated the crystal-induced injury. Inhibiting autophagy was also demonstrated to be an effective therapeutic approach through decreasing crystal deposition and oxalate-induced oxidative injuries in rat kidneys via inhibiting the activation of MAPK p38 signaling pathway (Duan et al., 2018). Lysosomal degradation is the last key process in the autophagic flux. In the present study, CTSD is a representative aspartic proteinase in lysosomes. A reduction in CTSD was found to inhibit the capacity of lysosomal degradation to mediate the blockade of autophagy in lead-induced nephrotoxicity (Song et al., 2017). The increased expression of CTSD in the kidneys and HK-2 cells exposed to oxalate stimulation observed in this study is consistent with the autophagic activity induced by crystals. Therefore, the augmenting CTSD levels in kidney and tubular cells was thought to enhance the injury by accelerating the autophagic flux. The administration of DS could exert the protective effects by inhibiting the enhanced autophagic activity in oxalate-induced renal injury (Figure 10). Luteolin, apigenin, and genistein are three popular herbal compounds to be investigated in DS. In the present study, luteolin, apigenin, and genistein exerted clear protective effects on the cell viability of NaOx-stimulated cells and were considered the active components of DS. Luteolin is a type of flavonoid found in DS. In previous research, luteolin was found to ameliorate cisplatin-induced nephrotoxicity (Domitrović et al., 2013) and LPS-induced renal injury (Xin et al., 2016) through the inhibition of oxidative stress, inflammation, and apoptosis in the kidneys. In this research, luteolin was demonstrated to protect the tubular cells by regulating the lysosomal activity though CTSD.

The occurrence of the epithelial mesenchymal transition of tubular cells and renal fibrogenesis induced by oxalate crystals has been demonstrated by multiple studies in mouse, rat, and cell models (Toblli et al., 2002; Convento et al., 2017). As our previous study showed, the expression of kidney interstitial markers (α-SMA and Vim) increased gradually in response to the glyoxylate-induced CaOx crystal formation and deposition (Hu et al., 2015). Fibronectin (FN1) is an extracellular matrix biomarker in the Type II epithelial-mesenchymal transition, which is induced in the context of inflammation and fibrosis (Carew et al., 2012). In the present research, the increase of fibronectin in the oxalate group implied the development of renal fibrosis. CTSD was also indicated to play an important role in renal fibrosis. The upregulated expression of CTSD was induced in a unilateral ureteric obstruction mice model. The inhibition of CTSD has been considered a new therapeutic approach to reduce renal fibrosis and slow the progression of CKD induced by obstructive nephropathy (Fox et al., 2016). Therefore, the potential role of CTSD in the renal fibrogenesis induced by oxalate crystals was proposed. DS and luteolin could attenuate crystalline nephropathy by inhibiting renal fibrosis.

As one of the inverse-docking targets, p38 MAPK and its signaling pathways have been indicated to be involved in diverse nephropathy including nephrolithiasis. Both oxalate and CaOx crystals have been demonstrated to selectively activate p38 MAPK in exposed tubular cells (Khan, 2004). The P38-MAPK signaling pathway has also been found to play important roles in pathological conditions associated with cellular apoptosis (Han et al., 2004) and the tight junction disruption in distal tubular epithelial cells following the deposition of CaOx (Yu et al., 2017). These associated pathological conditions and the deposition of CaOx could all be prevented by the inhibition of p38 MAPK. In addition, p38 MAPK has been shown to play a critical role in renal fibrosis based on a rat model of unilateral ureteric obstruction, and a blockade of p38 MAPK was considered a potential therapeutic option in the treatment of renal fibrosis (Stambe et al., 2004). In the present study, the induced phosphorylation level of p38 MAPK in the kidney tissue of mice was reversed by DS treatment. Therefore, p38 MAPK was considered a potential therapeutic target of DS in crystalline nephropathy. However, the active component of DS to target the MAPK P38 remains to be explored. Another differential protein, thrombospondin 1, abbreviated as THBS1 or TSP1, is an adhesive glycoprotein that can bind to fibronectin and collagens. THBS1 is also considered a cytokine that can promote the transcription of transforming growth factor-β (TGF-β) mRNA in the renal interstitial fibrosis of rats with a unilateral ureteral obstruction (Xie et al., 2008). In this study, the induction of THBS1 in the oxalate group indicated its potential role in nephrolithiasis. The blockade of THBS1-dependent TGF-β activity has been discovered to reduce proteinuria and improve markers of tubulointerstitial injury (fibronectin) in a murine model of diabetic nephropathy (Lu et al., 2011). THBS1 expression has also been found to be regulated by p38 MAPK in rat proximal tubular cells and mouse fibroblasts in response to TGF-β1 (Nakagawa et al., 2005). In this study, luteolin, apigenin, and genistein could protect oxalate-stimulated tubular cells by inhibiting the transcription of THBS1.

Cyclin-dependent kinase 2 (CDK-2) is a member of the cyclin-dependent kinase family of Ser/Thr protein kinases and involved in the control of the cell cycle. The increased activity of CDK-2 has been discovered in adverse renal diseases, such as renal failure during sepsis (Yang et al., 2009) and IgA nephropathy (Qiu et al., 2004). Cisplatin nephrotoxity was demonstrated to be dependent on CDK2 activity *in vivo* and *in vitro*. As an inhibitor of cdk2, p21 was found to protect renal cells from cisplatin cytotoxicity (Price et al., 2006). Inhibiting CDK2 activity has also been considered a potential therapeutic target for glomerular diseases characterized by podocyte proliferation (Griffin et al., 2005). In the oxalate group, CDK-2 has a trend of activation, which can be regulated by DS and luteolin. Further experiments are still needed to validate these trends.

Among the differential proteins, SLC5A2, SLC6A19, SLC12A1, and SLC22A12 are the ion transporters specifically or mainly expressed in the renal tubules. The reduction of these tubular transporters in the model group of the present study reflected the injury of renal tubules induced by oxalate crystals. Resulting from the dysfunctional expression of these carriers, the transport of some electrolytes and metabolites could be impaired. The concentrations of renal calcium and urinary calcium have been found to be significantly increased in oxalate-induced renal injuries (Brzica et al., 2013). The proteins from the family of annexins including ANXA1, ANXA2, ANXA3, and ANXA5 were all increased in the crystal group. In humans, annexins have been found not only inside cells, but also outside the cellular environment. ANXA1 and ANXA2 have been demonstrated to be markedly increased on the apical surface of high-calcium-exposed renal tubular cells as crystal adhesion molecules (Semangoen et al., 2008). Elevated ANXA5 levels have also been found in both acute and chronic renal conditions in accordance with their increase induced by oxalate crystals (Jeong et al., 2014).

In conclusion, the underlying action mechanism of DS in oxalate-induced kidney injury was explored in this study by combining proteomics analysis, network pharmacology prediction, and experimental validation. In addition, the potential targets (CTSD, p38 MAPK, and CDK2) and the active components (luteolin, apigenin, and genistein) of DS were successfully found based on this practical strategy.

## Author contributions

JH, WC, XD, and ZG conceived and designed the experiments. JH, HL, and SG performed the experiments. JH, HZ, and WL analyzed the data. WC, XD, and ZG contributed reagents, materials, analysis tools. JH, WC, and HL wrote the manuscript. All authors approved on the finally submitted version of the manuscript.

### Conflict of interest statement

The authors declare that the research was conducted in the absence of any commercial or financial relationships that could be construed as a potential conflict of interest.

## Abbreviations

CaOx: calcium oxalate
DS: *Desmodium styracifolium*
iTRAQ: isobaric tags for relative and absolute quantitation
MALDI-TOF-MS: matrix-assisted laser desorption ionization-time of flight-mass spectrometry
PPI: protein–protein interaction
CTSD: cathepsin D
MAPK: mitogen-activated protein kinase
CDK-2: cyclin-dependent kinase 2.
