# Supplementary material for: Exploring the Therapeutic Mechanism of Desmodium styracifolium on Oxalate Crystal-Induced Kidney Injuries Using Comprehensive Approaches Based on Proteomics and Network Pharmacology
Source: Front Pharmacol. 2018 Jun 13;9:620. doi: 10.3389/fphar.2018.00620 (PMC6008405; doi:10.3389/fphar.2018.00620)
Supplement: Supplementary file 1 [file Table_1.DOC]

Exploring the therapeutic mechanism of Desmodium styracifolium on oxalate crystal-induced kidney injury by comprehensive approaches based on proteomics and network pharmacology

Jiebin Hou ,Wei Chen, Hongtao Lu, Hongxia Zhao, Songyan Gao, Wenrui Liu, Xin Dong*, Zhiyong Guo*

*** Correspondence:** Xin Dong: dongxinsmmu@126.com，Zhiyong Guo：drguozhiyong@163.com.

# Supplementary Material and Methods

LC-MS/MS Analysis.

The dried pooled peptides were then reconstituted in A phase (20 mM ammonium formate solution, pH 10) for high-pH reverse-phase liquid chromatography (RPLC) separation, using Shimadzu LC -20AB HPLC system with an Ultremex SCX column (250 × 4.6 mm), with 0.8 mL/min of ﬂow rate. A total of 48 fractions were collected from 5min at per-min intervals and combined into new fractions according to parameters.

The combined fractions were dried, lyophilized, and reconstituted in 30 μL of 2% ACN-0.1% formic acid (FA) solution for nanoLC-MS/MS analysis. 4μl of the combined fractions were respectively analyzed on an eksigent nano LC-UltraTM system coupled with a TripleTOF^TM^5600 mass spectrometer (AB Sciex.USA). The samples were enriched using trap column（ChromXP C18_CL-3μm,120A,350μm *0.5μm）with mobile phase A(3ul/min,15min). At the same time, samples were loaded onto a C18 analytical column (0.075×150mm,3μm,120A) for reverse phase separation with 300 nl/min of flow rate. The following linear gradient was used: 0-0.1 min, 5-10% B（98% ACN，0.1% FA）;0.1-80 min,10-23% B; 80-105 min,23-35% B;105-106 min,35-80% B;106-110 min,80% B;110-110.5 min,80-5% B;110.5-120 min,5% B. The MS was operated in positive ion mode across the mass range of 350-1250m/z using 250 ms accumulation time per spectrum. The scan range of MS/MS was 100-1500 m/z including precursors with a charge state of +2 to +5.

Constituent compounds of DS

The constituent compounds of Desmodium styracifolium(DS) were selected according the previous references. The inclusion criteria for the compounds is to be mentioned at least 3 times in all the previous articles. As results, 15 most poplar compounds of DS were selected for further study.

# Supplementary Figures

**Supplementary Figure S1.** Differentially expressed proteins in models of HK-2 cell, mouse and rat.


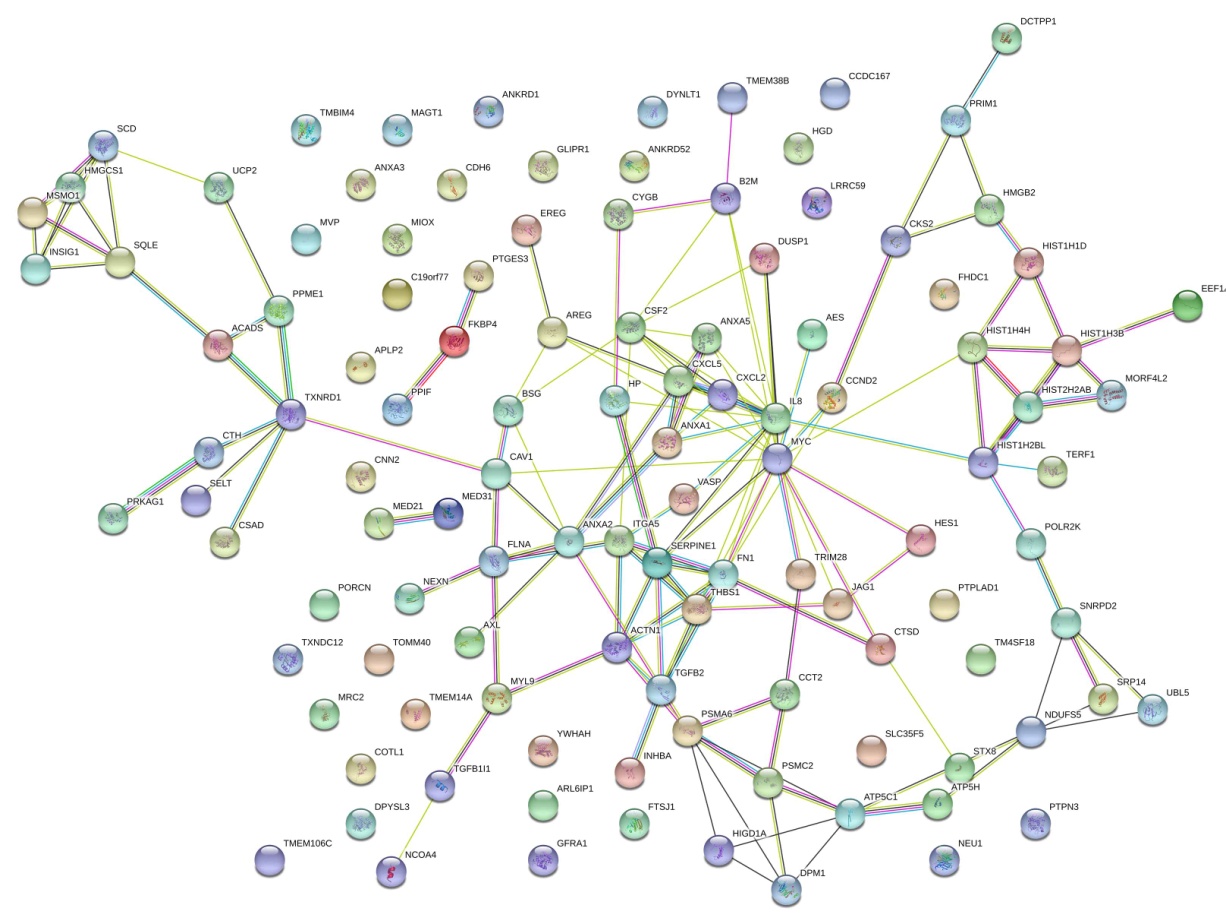


A


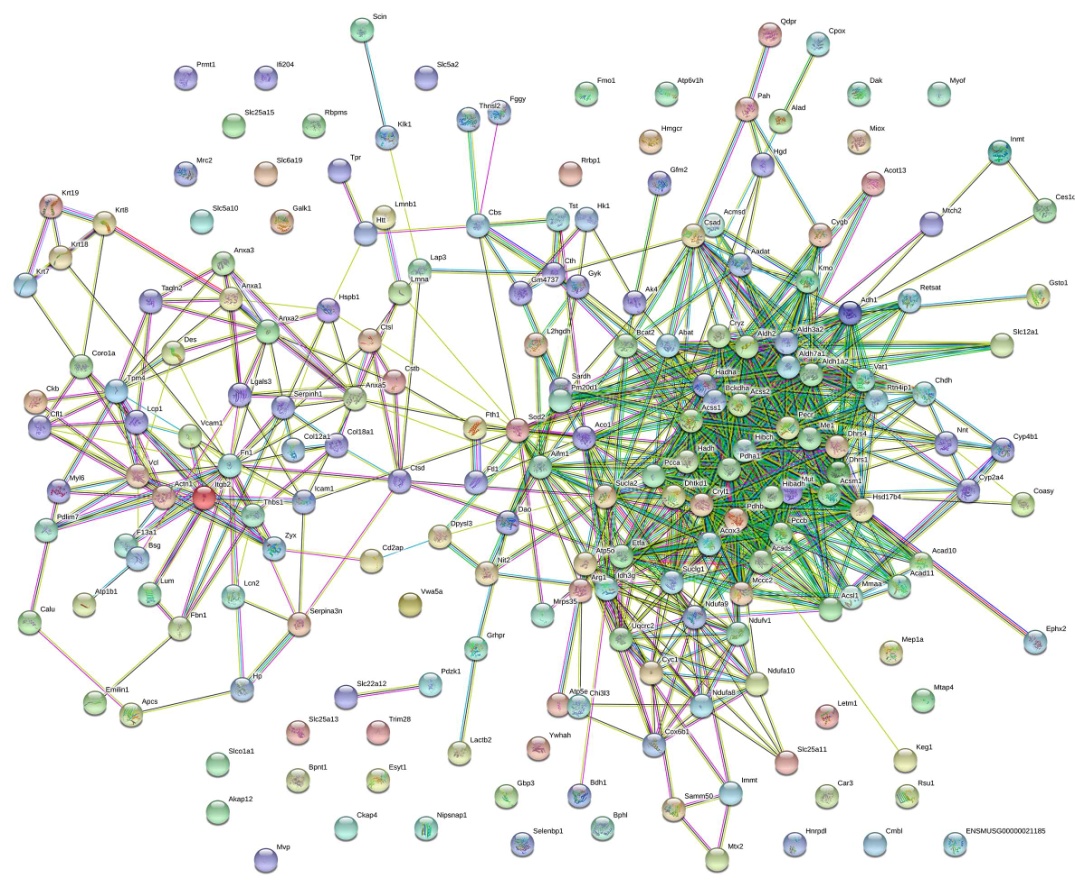


B

**
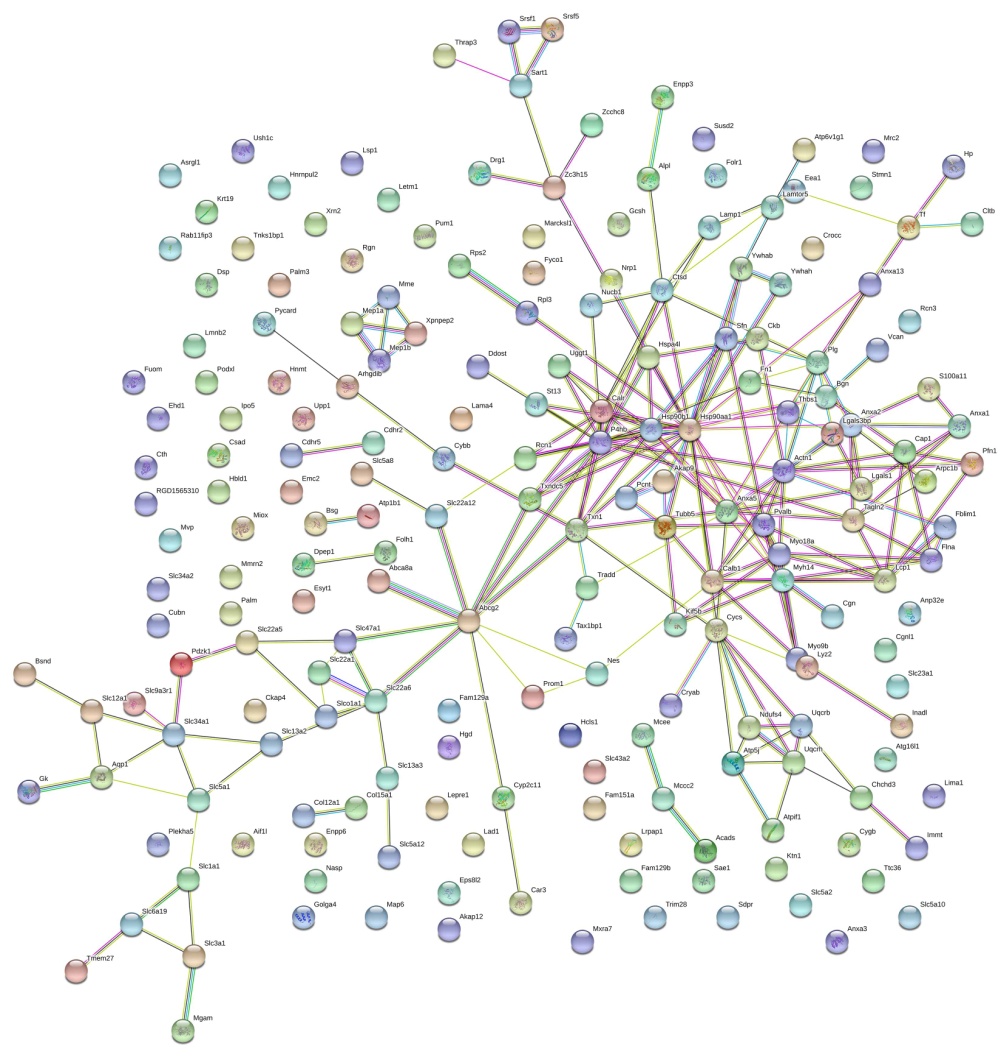
**

C

**Supplementary Figure S2.** PPI network of inverse docking targets with differential proteins.


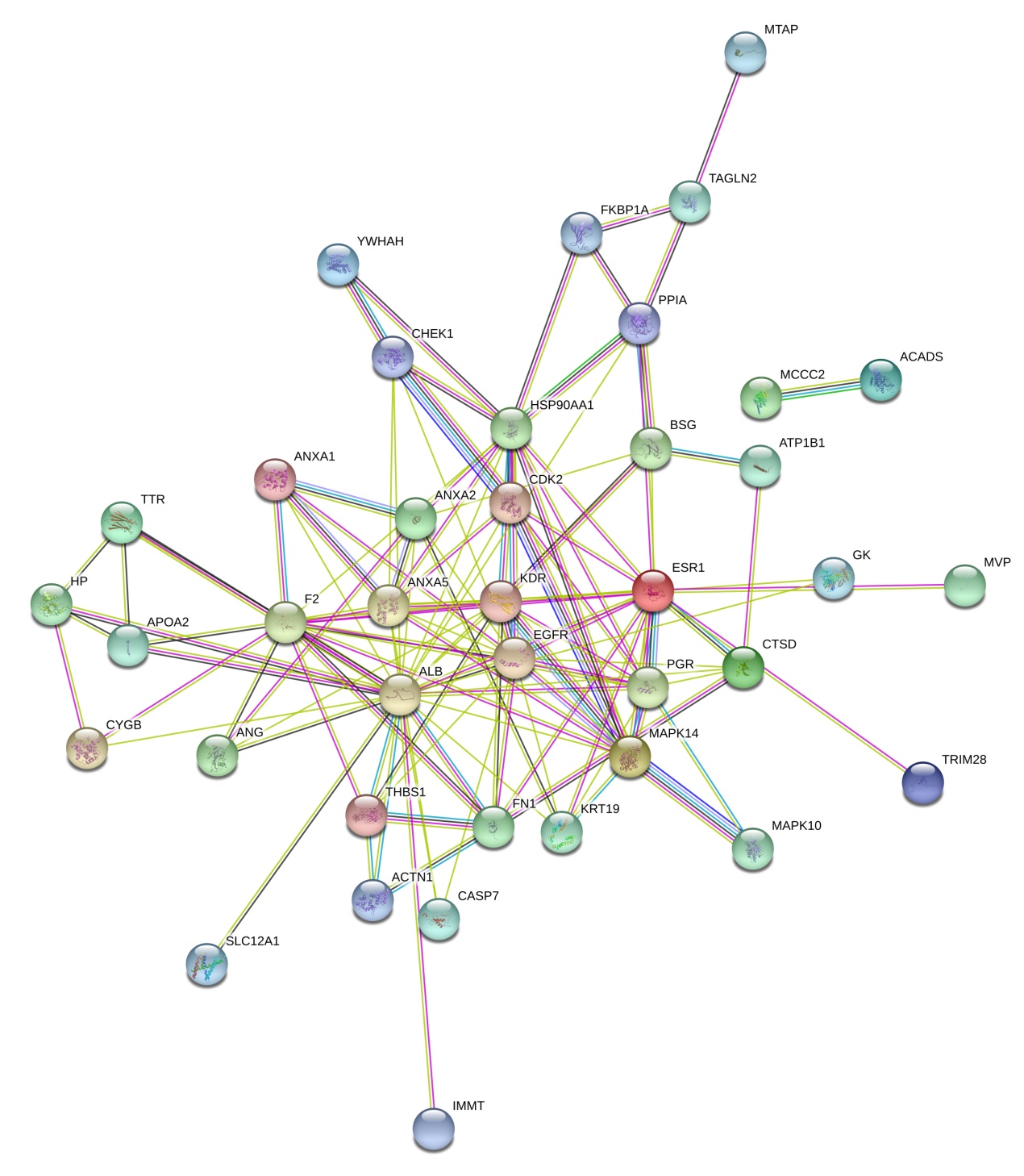


**Supplementary Figure S3.** Effects of luteolin, apigenin and genistein on mRNA levels in HK-2 cells. The mRNA levels of CTSD, THBS1 and ANXA5 in HK-2 cells were detected by real-time PCR. ^#^P < 0.05 versus untreated cells; *P < 0.05 versus cells treated with NaOx alone.

**
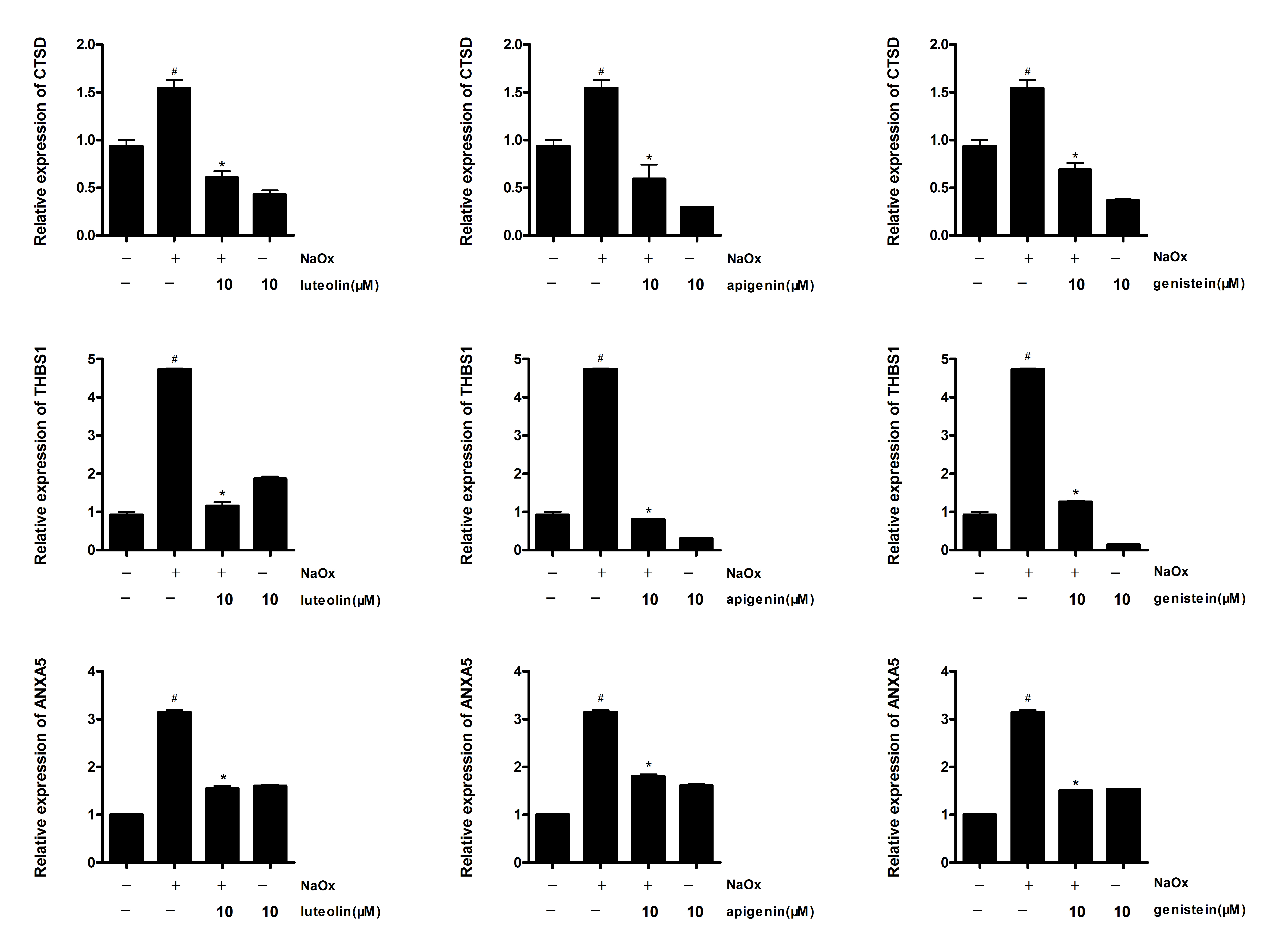
**

# Supplementary Tables

**Supplementary Table S1. The docking result of differential targets**

| Protein | PDB ID | Number | Ligand | Docking score |
| --- | --- | --- | --- | --- |
| YWHAH | 2C74 | 1 | Aromadendrin | 93.57 |
|  |  | 2 | Vicenin-3 | 138.07 |
|  |  | 3 | β-sitosterol | 113.53 |
|  |  | 4 | Schaftoside | 134.92 |
|  |  | 5 | Vicenin-2 | 130.27 |
|  |  | 6 | Homoferreirin | 106.00 |
|  |  | 7 | Formononetin | 95.12 |
|  |  | 8 | Apigenin | 97.04 |
|  |  | 9 | Luteolin | 99.22 |
|  |  | 10 | Chrysoeriol | 98.58 |
|  |  | 11 | 2-Hydroxygenistein | 91.34 |
|  |  | 12 | Isovitexin | 127.26 |
|  |  | 13 | Isoschaftoside | 126.46 |
|  |  | 14 | Genistin | 127.23 |
| ACTN1 | 2EYI | 1 | Aromadendrin | 93.91 |
|  |  | 2 | β-sitosterol | 80.58 |
|  |  | 3 | Homoferreirin | 90.67 |
|  |  | 4 | Apigenin | 80.86 |
|  |  | 5 | Luteolin | 85.33 |
|  |  | 6 | Isovitexin | 101.63 |
|  |  | 7 | Genistin | 98.31 |
| ANXA1 | 1AIN | 0 |  |  |
| ANXA2 | 1W7B | 1 | Genistin | 81.53 |
| ANXA3 | 1AII | 1 | Vicenin-3 | 95.90 |
|  |  | 2 | Schaftoside | 100.28 |
|  |  | 3 | Vicenin-2 | 101.61 |
|  |  | 4 | Apigenin | 80.08 |
|  |  | 5 | Luteolin | 81.30 |
|  |  | 6 | Isovitexin | 90.72 |
|  |  | 7 | Isoschaftoside | 95.73 |
|  |  | 8 | Genistin | 100.35 |
| ANXA5 | 1HAK | 1 | Aromadendrin | 93.03 |
|  |  | 2 | Vicenin-3 | 147.60 |
|  |  | 3 | β-sitosterol | 105.49 |
|  |  | 4 | Schaftoside | 137.70 |
|  |  | 5 | Vicenin-2 | 149.05 |
|  |  | 6 | Homoferreirin | 98.97 |
|  |  | 7 | Apigenin | 89.95 |
|  |  | 8 | Luteolin | 92.92 |
|  |  | 9 | Chrysoeriol | 95.95 |
|  |  | 10 | 2-Hydroxygenistein | 84.29 |
|  |  | 11 | Isovitexin | 104.26 |
|  |  | 12 | Isoschaftoside | 139.91 |
|  |  | 13 | Genistin | 118.98 |
| BSG | 3QQN | 1 | Aromadendrin | 89.72 |
|  |  | 2 | β-sitosterol | 98.43 |
|  |  | 3 | Homoferreirin | 98.14 |
|  |  | 4 | Formononetin | 82.49 |
|  |  | 5 | Apigenin | 89.90 |
|  |  | 6 | Luteolin | 94.59 |
|  |  | 7 | Chrysoeriol | 97.91 |
|  |  | 8 | 2-Hydroxygenistein | 83.16 |
|  |  | 9 | Isovitexin | 105.08 |
|  |  | 10 | Genistin | 115.68 |
| CTSD | 4OBZ | 1 | Aromadendrin | 97.80 |
|  |  | 2 | Vicenin-3 | 85.79 |
|  |  | 3 | β-sitosterol | 100.28 |
|  |  | 4 | Schaftoside | 80.33 |
|  |  | 5 | Vicenin-2 | 94.07 |
|  |  | 6 | Homoferreirin | 98.17 |
|  |  | 7 | Formononetin | 85.20 |
|  |  | 8 | Apigenin | 88.08 |
|  |  | 9 | Luteolin | 92.53 |
|  |  | 10 | Chrysoeriol | 91.14 |
|  |  | 11 | 2-Hydroxygenistein | 91.53 |
|  |  | 12 | Isovitexin | 112.73 |
|  |  | 13 | Isoschaftoside | 89.88 |
|  |  | 14 | Genistin | 113.45 |
| MRC2 | 5E4L | 1 | Aromadendrin | 90.62 |
|  |  | 2 | Vicenin-3 | 99.19 |
|  |  | 3 | β-sitosterol | 101.83 |
|  |  | 4 | Schaftoside | 104.69 |
|  |  | 5 | Vicenin-2 | 117.44 |
|  |  | 6 | Homoferreirin | 88.61 |
|  |  | 7 | Luteolin | 84.23 |
|  |  | 8 | Chrysoeriol | 81.11 |
|  |  | 9 | 2-Hydroxygenistein | 82.68 |
|  |  | 10 | Isovitexin | 94.76 |
|  |  | 11 | Isoschaftoside | 106.68 |
|  |  | 12 | Genistin | 96.98 |
| CTH | 5EIG | 1 | Vanillic acid | 80.62 |
| CSAD | 2JIS | 1 | Aromadendrin | 95.13 |
|  |  | 2 | Vicenin-3 | 129.92 |
|  |  | 3 | β-sitosterol | 107.82 |
|  |  | 4 | Schaftoside | 130.48 |
|  |  | 5 | Vicenin-2 | 132.02 |
|  |  | 6 | Homoferreirin | 92.08 |
|  |  | 7 | Apigenin | 80.78 |
|  |  | 8 | Luteolin | 92.58 |
|  |  | 9 | Chrysoeriol | 89.83 |
|  |  | 10 | 2-Hydroxygenistein | 80.82 |
| CYGB | 1URV | 1 | Aromadendrin | 89.28 |
|  |  | 2 | Vicenin-3 | 120.11 |
|  |  | 3 | β-sitosterol | 105.54 |
|  |  | 4 | Schaftoside | 100.23 |
|  |  | 5 | Vicenin-2 | 108.02 |
|  |  | 6 | Homoferreirin | 95.35 |
|  |  | 7 | Apigenin | 89.49 |
|  |  | 8 | Luteolin | 94.46 |
|  |  | 9 | Chrysoeriol | 93.33 |
|  |  | 10 | 2-Hydroxygenistein | 87.62 |
|  |  | 11 | Isovitexin | 115.84 |
|  |  | 12 | Isoschaftoside | 104.26 |
|  |  | 13 | Genistin | 121.25 |
| FN1 | 3M7P | 1 | Aromadendrin | 96.91 |
|  |  | 2 | Vicenin-3 | 112.16 |
|  |  | 3 | β-sitosterol | 104.51 |
|  |  | 4 | Homoferreirin | 94.31 |
|  |  | 5 | Formononetin | 95.04 |
|  |  | 6 | Apigenin | 82.67 |
|  |  | 7 | Luteolin | 81.66 |
|  |  | 8 | Chrysoeriol | 86.00 |
|  |  | 9 | Isovitexin | 88.15 |
| HP | 4X0L | 1 | Aromadendrin | 90.17 |
|  |  | 2 | Vicenin-3 | 109.48 |
|  |  | 3 | β-sitosterol | 98.46 |
|  |  | 4 | Homoferreirin | 90.05 |
|  |  | 5 | Formononetin | 89.05 |
|  |  | 6 | Apigenin | 92.17 |
|  |  | 7 | Luteolin | 102.93 |
|  |  | 8 | Chrysoeriol | 85.19 |
|  |  | 9 | 2-Hydroxygenistein | 90.47 |
|  |  | 10 | Isovitexin | 122.04 |
|  |  | 11 | Genistin | 108.51 |
| HGD | 1EYB | 1 | Vicenin-3 | 101.63 |
|  |  | 2 | Schaftoside | 94.44 |
|  |  | 3 | Vicenin-2 | 101.94 |
|  |  | 4 | Isoschaftoside | 95.87 |
| MIOX | 2IBN | 1 | Aromadendrin | 81.96 |
|  |  | 2 | Vicenin-3 | 112.60 |
|  |  | 3 | β-sitosterol | 103.93 |
|  |  | 4 | Schaftoside | 117.74 |
|  |  | 5 | Vicenin-2 | 109.82 |
|  |  | 6 | Isovitexin | 81.90 |
|  |  | 7 | Isoschaftoside | 122.79 |
|  |  | 8 | Genistin | 122.29 |
| MVP | 1Y7X | 1 | Aromadendrin | 103.90 |
|  |  | 2 | Vicenin-3 | 110.27 |
|  |  | 3 | β-sitosterol | 83.40 |
|  |  | 4 | Schaftoside | 110.94 |
|  |  | 5 | Vicenin-2 | 122.86 |
|  |  | 6 | Homoferreirin | 114.63 |
|  |  | 7 | Formononetin | 105.99 |
|  |  | 8 | Luteolin | 116.20 |
|  |  | 9 | 2-Hydroxygenistein | 110.99 |
|  |  | 10 | Isovitexin | 128.32 |
|  |  | 11 | Genistin | 116.21 |
| ACADS | 2VIG | 1 | Vicenin-3 | 114.90 |
|  |  | 2 | β-sitosterol | 118.03 |
|  |  | 3 | Vicenin-2 | 90.41 |
|  |  | 4 | Homoferreirin | 100.39 |
|  |  | 5 | Formononetin | 88.68 |
|  |  | 6 | Genistin | 109.83 |
| THBS1 | 2OUH | 1 | Vanillic acid | 91.19 |
|  |  | 2 | Aromadendrin | 107.49 |
|  |  | 3 | Vicenin-3 | 125.04 |
|  |  | 4 | β-sitosterol | 88.66 |
|  |  | 5 | Schaftoside | 81.14 |
|  |  | 6 | Vicenin-2 | 105.26 |
|  |  | 7 | Homoferreirin | 111.24 |
|  |  | 8 | Formononetin | 97.62 |
|  |  | 9 | Apigenin | 99.88 |
|  |  | 10 | Luteolin | 88.95 |
|  |  | 11 | Chrysoeriol | 108.28 |
|  |  | 12 | 2-Hydroxygenistein | 88.44 |
|  |  | 13 | Isovitexin | 105.99 |
|  |  | 14 | Genistin | 134.64 |
| TRIM28 | 2YVR | 1 | Vanillic acid | 83.40 |
|  |  | 2 | Aromadendrin | 88.25 |
|  |  | 3 | Homoferreirin | 96.22 |

**Supplementary Table S3.** The sequences of primers.

|  | Protein | Gene | Forward | Reverse |
| --- | --- | --- | --- | --- |
| 1 | Cathepsin D | CTSD | GCTGGACATCGCTTGCTGGATC | AGAGGCTGACGACGCTGACTG |
| 2 | Thrombospondin-1 | THBS1 | TTTGACATCTTTGAACTCACCG | AGAAGGAGGAAACCCTTTTCTG |
| 3 | Annexin A5 | ANXA5 | CAGAGGCACTGTGACTGACTTCC | TTCCTGGCGCTGAGCATTACTTC |

References:

Gao, R., and Guo, X. (2001). Separation and identification of chemical constituents of Desmodium styracifolium (in chinese). *Joumaj of Chinese Medicinal Material* 24**,** 724-725.

Lai, L., Lin, X., Chen, F., and Lai, X. (2016). Analysis of main active components in Desmodii Styraciflii Herba by HPLC-QTOF-MS and HPLC-DAD (in chinese). *Chinese Traditional and Herbal Drugs* 47**,** 3578-3585.

Li, X., Wang, H., Liu, G., Zhang, X., Ye, W., and Zhao, S. (2007). Study on chemical constituents from Desmodium styracifoliu (in chinese). *Joumaj of Chinese Medicinal Material* 30.

Liu, Z., Dong, Y., Wang, N., Wang, J., and Li, X. (2005). Chemical studies on the constituents of Desmodium styracifolium(Osb .)Merr (in chinese). *Journal of Shenyang Pharmaceutical University* 22**,** 422-424.

Meng, A. (2008). Research progress of medicinal plant Desmodium styracifolium (Osbeck) Merr. (in chinese). *Journal of Guangxi Academy of Science* 24**,** 148-151.

Su, W. (2014). *Isolation and purification of bioactive compounds from Desmodium styracifolium and Forsythia suspense via high-speed counter-current chromatography (in chinese).* Master, Central south university.

Su, W., Liu, Q., Yang, Q., Yu, J., and Chen, X. (2013). Separation and purification of four compounds from Desmodium styracifolium using off-line two-dimensional high-speed counter-current chromatography. *J Sep Sci* 36**,** 3338-3344.

Sun, D. (2013). *Comparation of main constituents in Herba Lysimachiae and Herba Desmodii Styracifolii and studies on pharmacokinetics and biliary excretion of Desmodii Styracifolii in rats by LC-MS/MS technologies (in chinese).* Master, Hebei medical university.

Sun, D., Dong, L., Guo, P., Shi, X., Gao, J., Ren, Y., Jiang, X., Li, W., Wang, C., and Wang, Q. (2013). Simultaneous detection of flavonoids and phenolic acids in Herba Lysimachiae and Herba Desmodii Styracifolii using liquid chromatography tandem mass spectrometry. *Food Chem* 138**,** 139-147.

Wang, Z., Bai, X., and Liu, F. (1998). Study on chemical constituents of Desmodium styracifolium (in chinese). *Journal of Guangxi Medical University* 15**,** 10-14.

Yang, J., Su, Y., and Wang, Y. (1993). Studies on the chemical constituents of Desmodium styracifolium (Osbeck) Merr. (in chinese). *Acta Pharmaceutica Sinica* 28**,** 197-201.

Zhou, C., Luo, J.G., and Kong, L.Y. (2012). Quality evaluation of Desmodium styracifolium using high-performance liquid chromatography with photodiode array detection and electrospray ionisation tandem mass spectrometry. *Phytochem Anal* 23**,** 240-247.
